# Pyrazine ring-based Na^+^/H^+^ exchanger (NHE) inhibitors potently inhibit cancer cell growth in 3D culture, independent of NHE1

**DOI:** 10.1038/s41598-020-62430-z

**Published:** 2020-04-02

**Authors:** Michala G. Rolver, Line O. Elingaard-Larsen, Anne P. Andersen, Laurent Counillon, Stine F. Pedersen

**Affiliations:** 1 Section for Cell Biology and Physiology, Department of Biology, Faculty of Science, University of Copenhagen, Copenhagen, Denmark; 2 Center for Medical Parasitology, Department of Immunology and Microbiology, Faculty of Health and Medical Sciences, University of Copenhagen, Copenhagen, Denmark; 3 Université Côte d’Azur, CNRS, France LP2M, 28 Avenue de Valombrose, and Laboratories of Excellence Ion Channel Science and Therapeutics, Nice, France

**Keywords:** Cancer, Small molecules, Breast cancer, Cancer, Chemotherapy, Cell biology, Cell death

## Abstract

The Na^+^/H^+^ exchanger-1 (NHE1) supports tumour growth, making NHE1 inhibitors of interest in anticancer therapy, yet their molecular effects are incompletely characterized. Here, we demonstrate that widely used pyrazinoylguanidine-type NHE1 inhibitors potently inhibit growth and survival of cancer cell spheroids, in a manner unrelated to NHE1 inhibition. Cancer and non-cancer cells were grown as 3-dimensional (3D) spheroids and treated with pyrazinoylguanidine-type (amiloride, 5-(*N*-ethyl-*N*-isopropyl)-amiloride (EIPA), 5-(*N*,*N*-dimethyl)-amiloride (DMA), and 5-(*N*,*N*-hexamethylene)-amiloride (HMA)) or benzoylguanidine-type (eniporide, cariporide) NHE1 inhibitors for 2–7 days, followed by analyses of viability, compound accumulation, and stress- and death-associated signalling. EIPA, DMA and HMA dose-dependently reduced breast cancer spheroid viability while cariporide and eniporide had no effect. Although both compound types inhibited NHE1, the toxic effects were NHE1-independent, as inhibitor-induced viability loss was unaffected by NHE1 CRISPR/Cas9 knockout. EIPA and HMA accumulated extensively in spheroids, and this was associated with marked vacuolization, apparent autophagic arrest, ER stress, mitochondrial- and DNA damage and poly-ADP-ribose-polymerase (PARP) cleavage, indicative of severe stress and paraptosis-like cell death. Pyrazinoylguanidine-induced cell death was partially additive to that induced by conventional anticancer therapies and strongly additive to extracellular-signal-regulated-kinase (ERK) pathway inhibition. Thus, in addition to inhibiting NHE1, pyrazinoylguanidines exert potent, NHE1-independent cancer cell death, pointing to a novel relevance for these compounds in anticancer therapy.

## Introduction

With more than 2 million estimated new cases in 2018, breast cancer remains the leading cause of cancer death in women globally^1^. While the luminal and HER2-enriched breast cancer subtypes can be treated with hormone receptor- and antibody-based targeted therapies, treatment of basal-like breast cancers still relies on chemotherapy^2^. In all subtypes, acquired or *de novo* treatment resistance remains a paramount obstacle^2^. The tumour microenvironment plays a central role in drug resistance, by limiting exposure of the tumour cells to anti-cancer drugs, and through selection for highly aggressive cancer cells^3,4^. A hallmark of the tumour microenvironment is profound acidity, caused by the high metabolic activity and increased acid extrusion of the rapidly growing tumour cells. Upregulation of acid extrusion is a ubiquitous characteristic of aggressive tumour cells, and we and others have shown that knockdown (KD) or genetic ablation of net acid-extruding transporters reduces tumour growth in several cancer models^5–11^. This renders inhibition of such transporters, alone or as combination therapy, a promising therapeutic approach, as suggested already decades ago^12^. The Na^+^/H^+^ exchanger isoform 1 (NHE1, SLC9A1) is a major regulator of intracellular pH (pH_i_) and is widely explored as a target in cancer as well as in other diseases (see^9,13^).

The first NHE1 inhibitors, still in widespread use, are derivatives of amiloride. These are termed pyrazinoylguanidine-type inhibitors as their core structure corresponds to that of amiloride, which is a pyrazinoylguanidine compound bearing a terminal acyl guanidine group at the 2-position and a Cl at the 6-position. The most commonly used pyrazinoylguanidine-type NHE1 inhibitors are 5-(*N*-ethyl-*N*-isopropyl) amiloride (EIPA), 5-(*N,N*-dimethyl) amiloride (DMA) and 5-(*N,N*-hexamethylene) amiloride (HMA). A second class of inhibitors with higher specificity for NHE1 feature replacement of the pyrazine core with a phenyl ring (benzoylguanidine class), with a variety of substituents at the 2- and 5-positions^13,14^. Widely used benzoylguanidine-type inhibitors are cariporide (HOE642) and eniporide^14,15^. Both compound classes inhibit NHE1 in part by competition with Na^+^ at the transport site, although other regions also contribute to inhibitor sensitivity^13,16,17^. Except for the pyrazinoylguanidine parent compound amiloride, inhibitors in both compound classes generally exhibit Ki values for NHE1 in the nanomolar range^14–16,18^.

The use of physiologically-relevant *in vitro* models can reduce costs and save animal lives by allowing three-dimensional (3D) *in vitro* drug efficacy screening prior to *in vivo* testing. Screening in 3D spheroids, which mimic tumour oxygen, pH- and nutrient gradients, as well as drug permeability and -response^3,19,20^, is a key element in studies of anticancer drugs^3,19,21,22^. Such studies are particularly important for drugs that are weak acids (cariporide, eniporide) and weak bases (pyrazinoylguanidines such as EIPA and amiloride), as pH will profoundly impact drug charge and hence distribution between cytosol, extracellular space, and acidic compartments^12,23^. Despite this, essentially all studies of NHE1 inhibitors in cancer cells were conducted under two-dimensional (2D) growth conditions which poorly reflect *in vivo* conditions^20^. Furthermore, several studies point to NHE1-independent effects of NHE1 inhibitors^24–30^, yet mechanistic insight into these effects is lacking.

The aim of this work was to assess the effects of pyrazinoylguanidine-type compared to benzoylguanidine-type NHE1 inhibitors or genetic ablation of NHE1, on growth, survival and sensitivity to anti-cancer therapy in various breast cancer subtypes grown as 3D spheroids. We found that 5-substituted pyrazinoylguanidine-type NHE1 inhibitors potently reduced the viability in MCF-7 and MDA-MB-231 spheroids. Notably, this effect was similar in wild type (WT) cells and after CRISPR/Cas9 knockout (KO) of NHE1. Both pyrazinoylguanidine- and benzoylguanidine-type NHE1 inhibitors inhibited NHE1 activity in 3D culture, yet the latter had no effect on viability. Loss of viability was generally, but not ubiquitously, greater in cancer cells than in non-cancer cells, and was associated with ER stress, autophagy inhibition, DNA damage, apoptosis, and paraptosis. The order of potency was HMA > EIPA > DMA > amiloride, with no detectable effects of the benzoylguanidines cariporide and eniporide. Accordingly, EIPA and HMA, but not cariporide, accumulated dramatically in the spheroids during long-term treatment, likely as a result of trapping in acidic compartments.

We conclude that pyrazinoylguanidine-type NHE1 inhibitors potently inhibit growth of cancer cell spheroids through multiple pathways and can do so independently of NHE1. We suggest these compounds may be useful in anticancer treatment.

## Results

### EIPA, but not cariporide, potently reduces cell viability in MCF-7 and MDA-MB-231 spheroids

**Figure 1:**
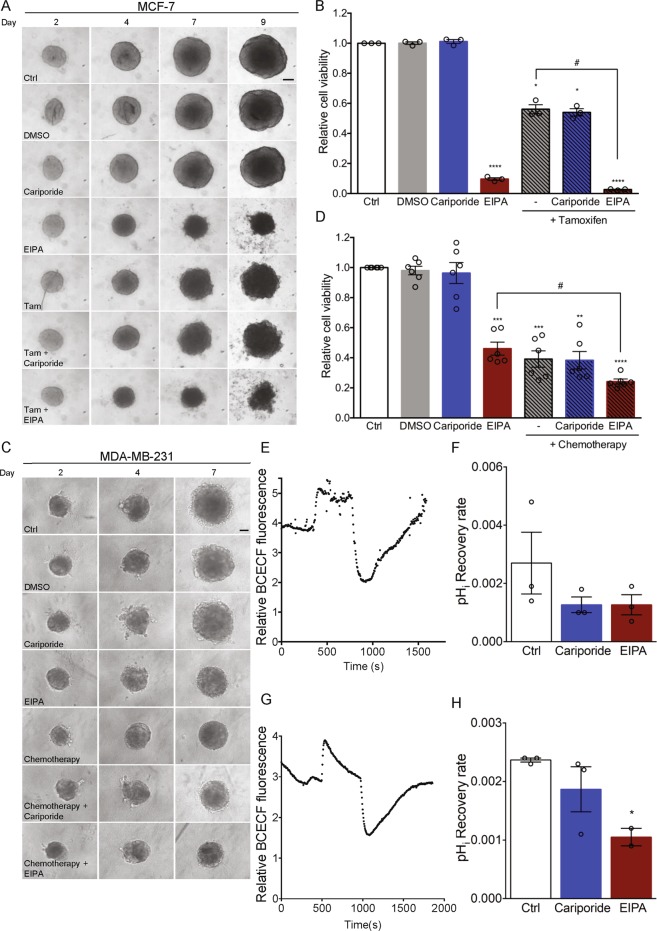
EIPA, but not cariporide, potently reduces viability of MCF-7 and MDA-MB-231 spheroids. MCF-7 and MDA-MB-231 spheroids were grown for 7–9 days. Spheroids were treated simultaneously with NHE1-inhibitors (cariporide (10 µM) or EIPA (10 µM)), breast cancer subtype specific anti-cancer therapy (tamoxifen (Tam, 2 µM) or chemotherapy (Cisplatin (18.75 nM), Doxorubicin (18.75 nM) and 5-FU (0.0625 nM)) or a combination thereof as indicated, on day 2 and 4 (MDA-MB-231) or 2, 4 and 7 (MCF-7). DMSO served as vehicle for the anti-cancer therapy. Light microscopic images of spheroids were acquired on day 2, 4, 7 (MDA-MB-231) and 9 (MCF-7). (**A**,**C**) Representative images of MCF-7 and MDA-MB-231 spheroids, respectively. 3–6 n. Scale bar: 100 µm. (**B**,**D**) Cell viability of MCF-7 (day 9) (**B**) and MDA-MB-231 (day 7) (**D**) spheroids, respectively. One-way ANOVA test with Tukey’s multiple comparisons post-test was used to determine statistically significant differences between treatment groups. * and # denotes significant differences between the treatment condition relative to DMSO and between two treatment conditions, respectively. For panel 1B, the *p*-value is 0.0116 and 0.0147 for control vs. tamoxifen + cariporide and tamoxifen vs. tamoxifen + EIPA, respectively, while for panel 1D the *p*-value is 0.0011 and 0.0143 for control vs. chemotherapy + cariporide and for EIPA vs. chemotherapy + EIPA, respectively. Error bars denote SEM. 3–6 n. (**E**,**G**) Representative traces demonstrating BCECF-fluorescence as a function of time during pH_i_ recovery of MCF-7 (day 4) (**E**) and MDA-MB-231 (day 4) (**G**) spheroids after NH_4_Cl pre-pulse. Fluorescence is depicted as the 485/440 nm ratio of BCECF intensity. 2–4 n (**F**,**H**) Quantification of pH_i_ recovery rate of MCF-7 (*p*-value = 0.343 for control vs. both cariporide and EIPA) (**F**) and MDA-MB-231 (*p*-value = 0.348 for control vs. cariporide, and 0.0461 for control vs. EIPA) (**H**) spheroids determined as the slope of the first 120 s after maximum acidification. Error bars denote SEM. 2–4 n. Statistical significance determined using a one-way ANOVA test with Tukey’s multiple comparisons test. 2–3 n.

Pharmacological inhibition of NHE1 using EIPA or cariporide sensitizes p95HER2-expressing MCF-7 human breast cancer cells grown in 2D culture to cisplatin (a purine crosslinker, which has an effect similar to that of DNA-alkylating agents) chemotherapy^31,32^. We therefore first asked whether NHE1 inhibitors can sensitize cancer cells to clinically relevant anticancer treatments. To maximize relevance to *in vivo* conditions, we grew cells as 3D spheroids, which mimic the tumour microenvironment and better model anticancer treatment response than 2D cultures^3,19,20,22^. Native MCF-7 cells - a model of luminal A breast cancer – were grown for 2 days as spheroids, followed by 7 days of treatment with the anti-oestrogen tamoxifen (2 µM), cariporide (10 µM), EIPA (10 µM), or a combination of tamoxifen and either inhibitor. The tamoxifen concentration was chosen based on a dose-response screen (Supplementary Fig. S1), and concentrations of cariporide and EIPA were chosen to ensure inhibition at the high Na^+^ concentration and serum content of growth medium, compared to the low Na^+^- and serum-free conditions used to determine Ki values. Spheroid growth was monitored by brightfield imaging (Fig. 1A), and a cell viability assay was performed on day 9 (Fig. 1B). As expected, 2 µM tamoxifen treatment resulted in spheroids with visibly frayed edges from day 7 and about 45% reduced cell viability at day 9 compared to untreated controls (*p* = 0.0181). Cariporide had no detectable effect on spheroid appearance or cell viability. In contrast, EIPA treatment resulted in small, irregular spheroids, and reduced day 9 viability to about 10% of that of control cells (*p* < 0.0001). Combination of tamoxifen and EIPA exacerbated spheroid disintegration and further reduced viability to less than 5% of that of controls (*p* < 0.0001). Propidium iodide (PI) staining was performed to visualize the spatial arrangement of cell death in the spheroids (Supplementary Fig. S2). PI staining was visible in spheroid cores only in control- and cariporide-treated spheroids, but throughout the spheroids after EIPA treatment, consistent with the near-complete loss of cell viability. Treatment with tamoxifen resulted in evenly distributed, sparse PI staining of the nearly disintegrated spheroids.

We next subjected human triple-negative breast cancer (TNBC) MDA-MB-231 cells to a chemotherapeutic regimen (18.75 nM cisplatin, 18.75 nM doxorubicin and 0.0625 nM 5-FU, doses chosen based on a pilot dose-response screen, Supplementary Fig. S1). This combination mimics the clinical TNBC-treatment termed CAF, except that we used another alkylating agent, cisplatin, to replace cyclophosphamide, which has to be metabolized in the liver for conversion to its active DNA-alkylating form and therefore cannot be used in cell culture. Compared to untreated controls, viability was reduced to about 45% by the chemotherapeutic regimen (*p* = 0.0009), by about 50% by 10 µM EIPA (*p* = 0.0005), and further reduced, to about 25%, by the combination of chemotherapy and EIPA (*p* < 0.0001), whereas cariporide had no effect (Fig. 1C,D).

Importantly, both EIPA and cariporide inhibited recovery of pH_i_ following an acid load in MCF-7 (Fig. 1E,F) and MDA-MB-231 spheroids (Fig. 1G,H) as well as in 2D culture (Supplementary Fig. S3). The dramatically different effects of EIPA and cariporide on cell viability thus did not reflect different capacity for inhibition of NHE1 activity at the doses used. In MDA-MB-231 spheroids, cariporide appeared less potent than EIPA in inhibiting pH_i_ recovery. Given the much tighter architecture of MCF-7- compared to MDA-MB-231 spheroids^33^, it seems unlikely that cariporide penetrated the former more effectively than the latter, and we suggest that the apparent lower potency of cariporide in MDA-MB-231 cells reflects the upregulation of other net acid extrusion pathways in these cells during spheroid growth (see Discussion).

Collectively, these results show that Luminal A and TNBC breast cancer cells grown as 3D spheroids exhibit remarkably reduced cell viability upon long-term treatment with the pyrazinoylguanidine-type NHE1 inhibitor EIPA, but not with the benzoylguanidine-type inhibitor cariporide. This effect is partially additive to that of clinically relevant chemotherapy and does not reflect differential effects of the compounds on NHE1 activity.

### EIPA-induced loss of viability is cell-type specific and most pronounced in cancer cells

**Figure 2:**
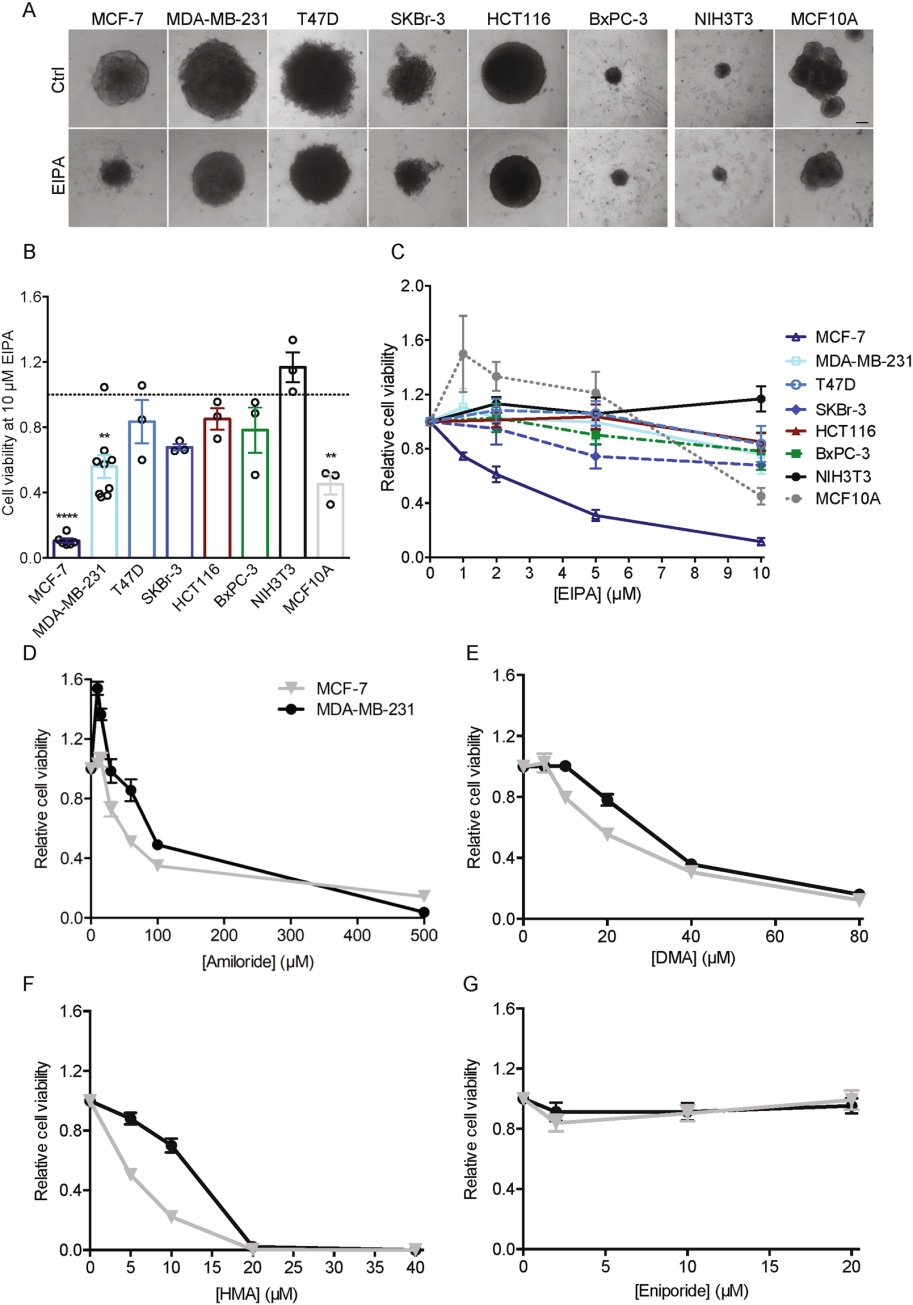
Pyrazinoylguanidine cytotoxicity is cell type-dependent and most potent for EIPA and HMA. Spheroids of the human cancer cell lines HCT116, BxPC-3, T47D, SKBr-3, MDA-MB-231 and MCF-7 (colon, pancreatic and 4 different breast cancer subtypes, respectively), the non-tumorigenic mammary epithelial cell line, MCF10A and the murine fibroblast cell line, NIH3T3, were grown for 9 days. Spheroids were treated with EIPA (0–10 µM) on day 2, 4, and 7. Spheroid growth was monitored by light microscopic imaging and a viability assay was performed on day 9. (**A**) Representative images of control- and EIPA- (10 µM) treated spheroids on day 9. Scale bar: 100 µm. Data are from 3 independent experiments. (**B**) Bar plot displaying cell viability at 10 µM EIPA on day 9 for each cell line. Error bars denote SEM. Data are from 3–9 independent experiments per condition. Dotted grey line represents control spheroids. Statistical significance determined using one-way ANOVA with Dunnett’s multiple comparisons test. **Denote p = 0.0020 for MDA-MB-231 and 0.0017 for MCF10A, while **** denote p < 0.0001 for MCF-7. Values from Fig. 1B,D are included for MCF-7 and MDA-MB-231. (**C**) Cell viability as a function of EIPA concentration on day 9. Error bars denote SEM. Data are from 3 independent experiments. (**D**–**G**) MCF-7 and MDA-MB-231 were grown for 9 days and treated with increasing doses of the respective NHE1 inhibitors amiloride (0–500 µM) (**D**), DMA (0–80 µM) (**E**), HMA (0–40 µM) (**F**) and eniporide (0–20 µM) (**G**) on day 2, 4 and 7. Graphs display day 9 viability. Error bars denote SEM. Data are from 3 independent experiments.

To determine whether the dramatic effect of EIPA on 3D growth was cancer cell specific, we cultured a series of cancer- and non-cancer cell lines as spheroids and exposed them to EIPA (0–10 µM) for 7 days (Fig. 2A–C). The cell lines investigated were: T47D (breast cancer, luminal A), SKBr-3 (breast cancer, HER2-positive), HCT116 (colon cancer), BxPC-3 (pancreatic cancer), MCF10A (non-tumorigenic mammary epithelial cells), and NIH3T3 (mouse fibroblasts). In the cancer cells, the greatest loss of viability was seen for MCF-7 cells, followed by MDA-MB-231, BxPC-3 and SKBr-3 cells. Growth of non-carcinogenic cells was less affected by EIPA: spheroids of NIH3T3 fibroblasts were fully unaffected up to 10 µM, the highest concentration used, whereas viability of MCF10A spheroids was if anything increased by EIPA at concentrations up to 5 µM, yet decreased at 10 µM EIPA (Fig. 2A–C). Cariporide at 10 µM had no detectable effect on spheroid viability for any of the cell lines tested (Supplementary Fig. S4).

These results show that EIPA cytotoxicity is cell type dependent, with strong loss of viability in some breast cancer subtypes, while other cell types are less or not detectably affected by EIPA.

### Cytotoxic effects of other NHE1 inhibitors in MCF-7 and MDA-MB-231 spheroids

To understand which functional groups are associated with cytotoxicity of NHE1 inhibitors, we next compared the ability of various pyrazinoylguanidine- and benzoylguanidine-type NHE1 inhibitors to induce cell death in MCF-7 and MDA-MB-231 spheroids. Table 1 shows the chemical structures and estimated dose resulting in a 50% reduction in cell viability (EC_50_) compared to reported 50% inhibitory concentration (IC_50_) values for NHE1 inhibition and the lipophilicity (LogD and/or LogP values). Ki represents the inhibition constants for NHE1 activity, as reported in the literature. Figure 2D–G shows corresponding dose-response curves for amiloride, DMA, HMA, and eniporide. For MCF-7 spheroids, the order of cytotoxicity was HMA > EIPA > DMA > amiloride, with an EC_50_ of HMA of only 2.5 µM. MDA-MB-231 cells were generally less sensitive than MCF-7 cells, but the order of cytotoxicity was similar. Neither cariporide nor eniporide, another benzoylguanidine-type compound with a reported IC_50_ for NHE1 of only 4.5 nM, had detectable effects on spheroid viability.

**Table 1:** Effect of pyrazineguanidine and benzoylguanidine compounds on viability of MCF-7 and MDA-MB-231 spheroids.

	Structure^a^	Lipophilicity^b^	Reported Ki values for NHE1 (µM)^c^	Reported IC_50_ values for NHE1 (µM)^d^	Experimentally determined EC_50_ values for compound-induced loss of viability (µM)^e^			
					MCF-7	95% CI	MDA-MB-231	95% CI
Amiloride		0.68 (LogD) 0.93 (LogP)	1.0–7.0	3.7–60	30.5	18.23–42.28	55.8	32.51–81.68
DMA		1.64 (LogD)	0.023–0.5	0.19	16.6	8.47–32.41	59	30.43–114.20
EIPA		3.2 (LogD)	0.01–0.1	0.016–0.8	4.8	1.29–17.78	19.6*	-
HMA		2.94 (LogD)	0.013	—	2.5	0.86–7.33	13.5	4.73–38.34
Cariporide		−1.01 (LogP)	0.008–0.04	0.03–3.4	ND**	ND	ND**	ND
Eniporide		−1.03 (LogP)	—	0.0045–0.38	ND***	ND	ND***	ND

The table displays reported IC_50_ and Ki values for NHE1 for the tested inhibitors, alongside experimentally obtained EC_50_ values for compound-induced loss of viability. ^a^Chemical structure of inhibitors. Blue: pyrazine ring characteristic for pyrazineguanidine compounds. Green: phenyl ring characteristic for benzoylguanidine compounds. Red highlights the different substitutions on the 5-position. ^b^The reported lipophilicity of each compound indicated by LogD and/or LogP values. ^c^Reported NHE1 Ki values presented in µM. ^d^Reported NHE1 IC_50_ values, determined in the presence of 100–130 mM Na^+^, presented in µM. ^e^Experimentally obtained EC_50_ values based on 3 n for each inhibitor. EC_50_ values were calculated in GraphPad Prism by nonlinear regression using the equation Y = Bottom + (Top-Bottom)/(1 + 10^((X-logIC_50_))), where X is the logarithm of the inhibitor concentration, top and bottom are the highest and lowest cell viability responses measured, respectively, and Y is the relative cell viability. The corresponding 95% confidence intervals for each EC_50_ value is presented alongside. ND: not determined. *EC_50_ calculated from trendline equation generated in Excel. **No effects of concentrations up to 10 µM. ***No effect of concentrations up to 20 µM. Table refs. ^13,15,17,42,48–51,56–61^.

These results demonstrate that pyrazinoylguanidine-type NHE1 inhibitors potently reduce viability in MCF-7 and MDA-MB-231 spheroids, whereas benzoylguanidine-type compounds have no detectable effect. As both classes of compounds inhibit NHE1 activity in these spheroids to a similar extent, and reported Ki values for NHE1 are lowest for the benzoylguanidines (Fig. 1E–H, Table 1), these results indicate that the effects on viability are NHE1-independent.

### The EIPA-mediated cytotoxicity of MCF-7 and MDA-MB-231 cells is NHE1-independent

**Figure 3:**
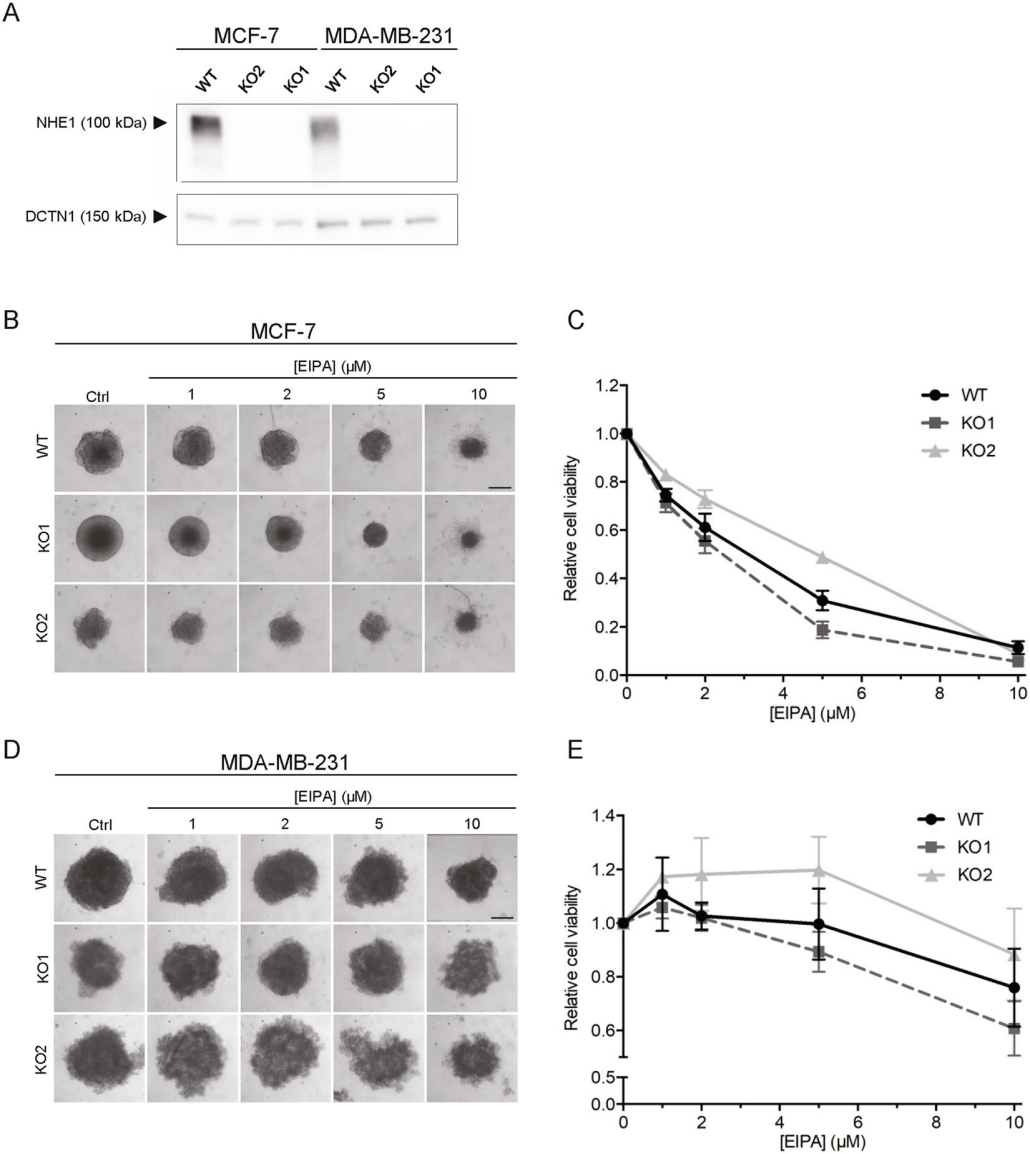
EIPA-mediated cytotoxicity is NHE1-independent. Spheroids of MCF-7 and MDA-MB-231 WT and two NHE1 CRISPR/Cas9 KO cell lines for each subtype were grown for 9 days and treated with EIPA (0–10 µM) on day 2, 4, and 7. Spheroid growth was monitored by light microscopic imaging and cell viability was assessed on day 9. (**A**) Representative Western blots of MCF-7 and MDA-MB-231 WT and KO cells (2D) blotted with an antibody directed against NHE1. Dynactin **(**DCTN1) serves as loading control. Full length blots are presented in Supplementary Fig. S6. (**B**,**D**) Representative images of MCF-7 (**B**) and MDA-MB-231 (**D**) WT and KO spheroid size on day 9. Scale bar: 200 µm. (**C**,**E**) Cell viability of MCF-7 (**C**) and MDA-MB-231 (**E**) WT and KO spheroids on day 9. 3 n. WT measurements are identical to the ones presented in Fig. 2C.

To conclusively determine whether the cytotoxic effects were NHE1-independent, MCF-7 and MDA-MB-231WT cells and two NHE1 CRISPR/Cas9 KO clones for each cell type^33^ were grown as spheroids and subjected to EIPA (0–10 µM) as above (Fig. 3). Notably, the effect of EIPA on viability was identical in WT and NHE1 KO spheroids, for both MCF-7 and MDA-MB-231 cells. Similarly, the response of MCF-7 NHE1 shRNA KD spheroids (~50% reduced NHE1 expression compared to vector controls^33^) to cariporide, EIPA, and tamoxifen treatment was essentially identical to that of WT MCF-7 cells (compare Fig. 1 to Supplementary Fig. S5).

These results demonstrate that the cytotoxic effect of EIPA is independent of NHE1.

### Pyrazinoylguanidine NHE1 inhibitors accumulate intracellularly in breast cancer cell spheroids

**Figure 4:**
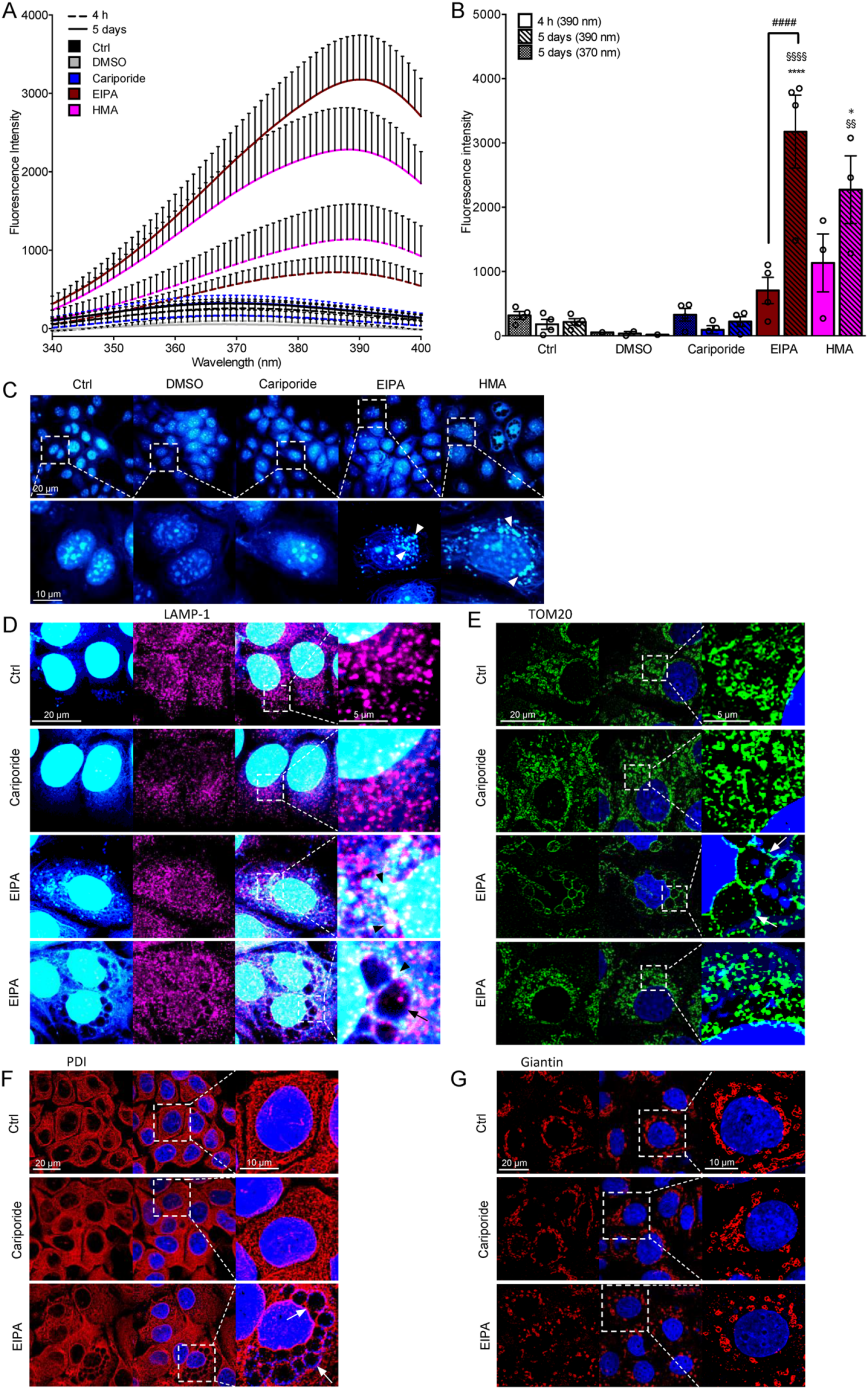
The pyrazinoylguanidine NHE1 inhibitors EIPA and HMA accumulate intracellularly in MCF-7 spheroids and monolayer cultures. MCF-7 spheroids grown for 2 or 7 days were treated with cariporide (10 µM), EIPA (10 µM) or HMA (10 µM) for 4 h or 5 days, respectively. DMSO served as a vehicle for HMA. Inhibitor fluorescence intensity was measured for three individual spheroids per condition. (**A**) Traces of an excitation scan from 340–400 nm. Emission was recorded at 420 nm. Error bars denote SEM. 3–4 n (except for DMSO which is 1–2 n). (**B**) Quantification of fluorescence intensity. A one-way ANOVA with Tukey’s multiple comparisons test was used for testing statistical significance between conditions. * and **** denote *p* = 0.0353 and <0.0001, respectively, between treatment and their respective control; ^####^denotes p < 0.0001 between 4 h and 5 days (390 nm) of the same condition, §§ denotes *p* = 0.0016 between cariporide and HMA, and §§§§ denotes *p* < 0.0001 between cariporide and EIPA. Error bars denote SEM. 3–4 n (except for DMSO which is 1–2 n). It should be noted that the fluorescence intensities have not been corrected for spheroid volume, and since HMA- and EIPA-treated spheroids are much smaller on day 7 than control- and cariporide-treated spheroids, the increase in fluorescence is substantially under-estimated. (**C**) Immunofluorescence images and zooms (white boxes) of MCF-7 cells treated with cariporide (10 µM), EIPA (10 µM) or HMA (10 µM) for 48 h. Arrow heads denote accumulated inhibitor. Representative of 3–4 independent experiments. Scale bars: 20 µm, 10 µm (zoom). (**D**–**G**) Immunofluorescence images and zooms (white boxes) of cariporide- (10 µM) and EIPA- (10 µM) treated (4 days) MCF-7 cells. Images are representative of 3 independent experiments. Arrows denote vacuoles. **D)** LAMP-1. Arrow heads indicate co-localization of EIPA and LAMP-1. Scale bars: 20 µm, 5 µm (zoom). (**E**) TOM20. Scale bars: 20 µm, 5 µm (zoom). (**F**) Giantin. Scale bars: 20 µm, 10 µm (zoom). **G)** PDI. Scale bars: 20 µm, 10 µm (zoom). Image overlays and adjustments of the intensities were performed using ImageJ software.

Amiloride and its derivatives are weak bases, with reported pK_a_ values ranging between 8.5 and 8.7 for amiloride, DMA, HMA, and EIPA^15,34^. In contrast, cariporide and eniporide are weak acids, with pK_a_ values of around 6.2 and 6.0, respectively^35,36^. We therefore speculated that the cytotoxicity of pyrazinoylguanidine-type NHE1 inhibitors might reflect their accumulation in acidic organelles during spheroid growth. To investigate this, we exploited the intrinsic fluorescence properties of these compounds^37,38^ and measured the fluorescence emission at 420 nm after excitation at 340–400 nm. Spheroids were grown for two days, treated with cariporide, EIPA or HMA (all at 10 µM), and accumulation examined after 5 days in continued presence of the inhibitor, compared to 4 h after inhibitor addition (when the compounds have permeated the spheroids but accumulation is expected to be minimal). Compared to control- and cariporide-treated spheroids, spheroids treated with EIPA or HMA displayed a markedly higher intrinsic fluorescence already after 4 h of treatment, with peak fluorescence intensity observed at around 390 nm (Fig. 4A,B). The absolute fluorescence intensity at this wavelength, quantified over multiple experiments, increased significantly from 4 h to 5 days of treatment for EIPA- and HMA-treated spheroids - despite their smaller volume (caused by inhibitor-induced loss of viability). In marked contrast, control- and cariporide-treated spheroids exhibited no detectable change in intrinsic fluorescence, despite the similar spectral properties (Fig. 4A).

To assess where in the cells the compound accumulation occurred, cells in 2D culture were treated with EIPA (10 µM) or HMA (10 µM) for 2–4 days, fixed and their intrinsic fluorescence analysed by UV illumination (Fig. 4C). Compound accumulation was detectable as the appearance of distinct, primarily punctate structures in the perinuclear region, which were never observed in control- or cariporide-treated cells. The punctate accumulation overlapped partially, but not completely, with staining for the lysosomal marker lysosomal-associated membrane protein 1 (LAMP-1) (Fig. 4D, arrow heads). No marked changes in lysosomal organization were detectable. In some cells, EIPA as well as HMA treatment induced the appearance of large perinuclear vacuolar structures (Fig. 4D–F, arrows). This pattern of vacuolization is characteristic of the cell death process known as paraptosis, characterized by damage to, and dilation of mitochondria and endoplasmic reticulum (ER)^39,40^. We therefore assessed the status of mitochondria, ER and Golgi by immunofluorescence analysis of their respective markers, mitochondrial import receptor subunit TOM20, protein disulfide isomerase (PDI), and giantin. EIPA treatment was associated with extensive mitochondrial fragmentation (Fig. 4E), intracellular vacuolization and a diminished Golgi area (Fig. 4G). The vacuoles appeared to be “pushing” on the nucleus (Fig. 4D–F) and surrounded by mitochondrial membrane (Fig. 4E) and ER (Fig. 4F).

These results show that EIPA and HMA undergo marked accumulation in cancer cell spheroids. Accumulation appears to be primarily lysosomal, and is associated with extensive perinuclear vacuolization, mitochondrial fragmentation, and ER reorganization.

### EIPA and HMA treatment elicit DNA damage, ER stress, mitochondrial fragmentation, autophagic arrest, and PARP cleavage

**Figure 5:**
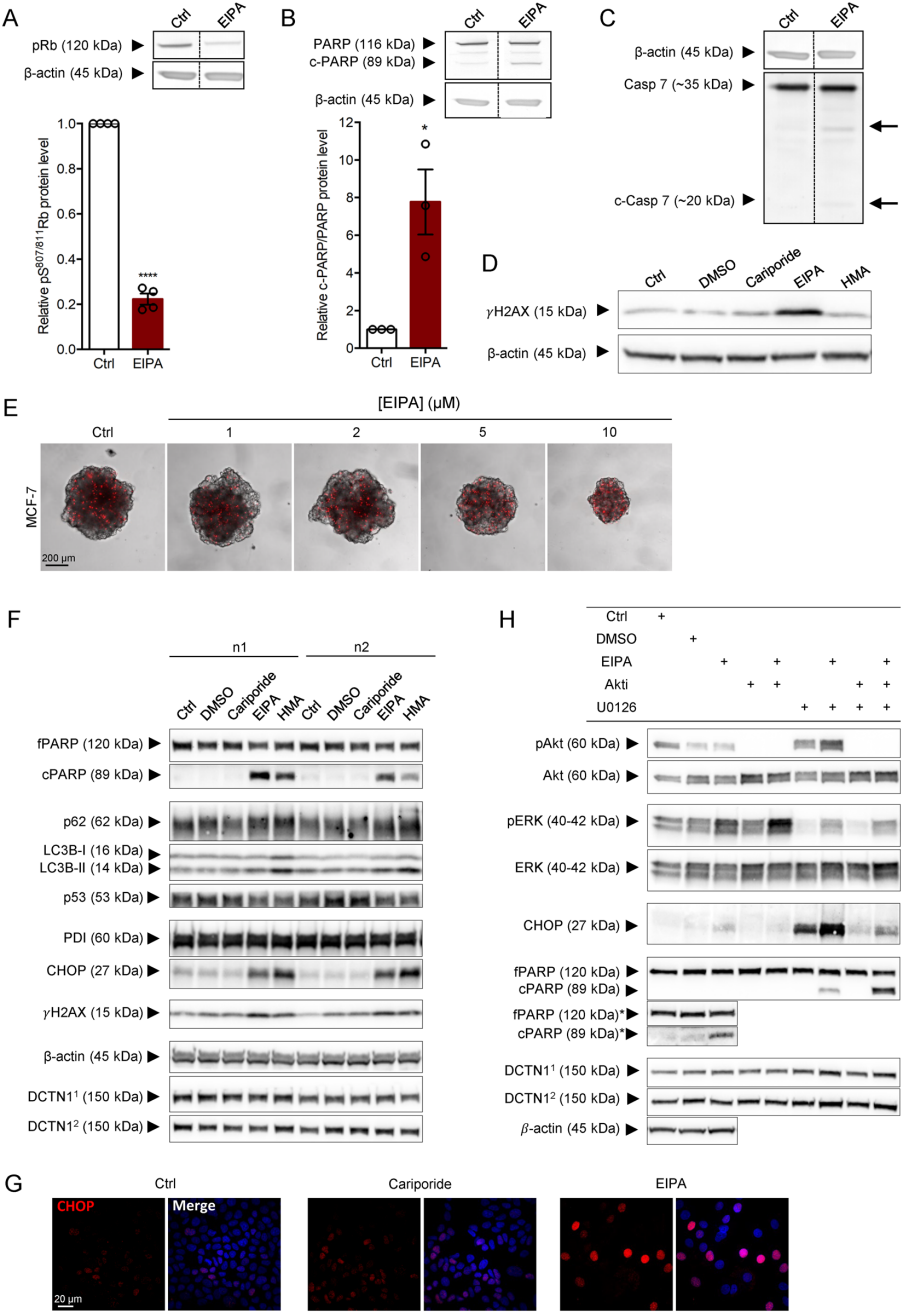
EIPA-mediated cytotoxicity involves DNA-damage, ER-stress, dysregulation of autophagy and reduced proliferation. (**A**–**C**) Top panel: Representative Western blots of MCF-7 spheroids treated with EIPA (10 µM) for 48 h. Lower panel: Quantification of band intensities normalized to control. (**A**) Retinoblastoma protein (pRb) phosphorylation (S807/811). 4 n; (**B**) PARP cleavage. 3 n; (**C**) Caspase 7 cleavage. (MCF-7 cells do not express caspase 3). 3 n. Error bars denote SEM. A paired (**A**) or ratio paired t-test (**B**) was used to test for statistically significant differences between conditions. (**D**) Representative Western blot of MCF-7 spheroids treated with cariporide (10 µM), EIPA (10 µM) or HMA (10 µM) on day 2 and 4. Lysates were prepared after 7 days of growth and blotted for γH2AX. 3 n. (**E**) PI-stained MCF-7 spheroids grown for 9 days and treated with EIPA (0–10 µM) on day 2, 4 and 7. Representative of 3 independent experiments. Scale bar: 200 µm. Image overlays and adjustments of the intensities were performed using ImageJ software. (**F**) Representative Western blots of MCF-7 cells treated with cariporide (10 µM), EIPA (10 µM) or HMA (10 µM). DMSO serves as a vehicle for HMA. β-actin: loading control for CHOP and PDI; DCTN1^1^: loading control for p53 and γH2AX; DCTN1^2^: loading control for fPARP, cPARP, p62 and LC3B. (**G**) CHOP-stained MCF-7 cells treated with cariporide (10 µM) or EIPA (10 µM) for 4 days. Representative images of 3 independent experiments. Scale bar: 20 µm. Image overlays and adjustments of the intensities were performed using ImageJ software. (**H**) Representative Western blots of MCF-7 cells treated with EIPA (10 µM), Akti (1 µM), U0126 (10 µM) or a combination thereof. DMSO serves as a vehicle for Akti and U0126. *The Ctrl, DMSO and EIPA lanes were additionally run on a separate gel to allow longer exposure to visualize cPARP. β-actin: loading control for fPARP* and cPARP*. DCTN1^1^: loading control for pERK, ERK, fPARP and cPARP; DCTN1^2^: loading control for pAkt, Akt and CHOP. fPARP: full length PARP; cPARP: cleaved PARP. Full length blots are presented in Supplementary Fig. S6.

To gain insight into the mechanisms underlying the cytotoxic effects of the pyrazinoylguanidines, MCF-7 spheroids were lysed 48 h after treatment with 10 µM EIPA, and immunoblotted for phosphorylated retinoblastoma protein (pRb), Poly-ADP Ribose Polymerase (PARP) and cleavage of caspase-7 (the main apoptotic effector caspase in MCF-7 cells, which lack caspase-3). This revealed that EIPA treatment elicited a 5-fold reduction in pRb level (*p* < 0.0001), an 8-fold increase in the c-PARP/PARP ratio (*p* = 0.0132), and slight caspase-7 cleavage (Fig. 5A–C). Some NHE1 inhibitors have been proposed to intercalate in DNA and cause DNA damage^41^. In agreement with this, Histone 2AX phosphorylation (γH2AX) was increased in EIPA-treated spheroids (Figs. 5D, 5 days of treatment). Finally, with increasing EIPA concentrations, PI-permeable cells were distributed throughout the spheroids rather than restricted to the hypoxic core as in controls (Fig. 5E).

To allow more detailed subcellular analysis, MCF-7 cells were next grown as monolayers for 4 days under control conditions or in presence of 10 µM EIPA or HMA, and subjected to immunofluorescence analysis and Western blotting. Similar to the results obtained in 3D spheroids, EIPA and HMA exerted potent cytotoxic effects, while there was no detectable toxicity of cariporide (Fig. 5F). Both EIPA and HMA elicited PARP cleavage and γH2AX upregulation, but not p53 upregulation (Fig. 5F). Pyrazinoylguanidine treatment also induced marked nuclear upregulation of the ER stress-induced transcription factor C/EBP Homologous Protein (CHOP (also known as GADD153)) (Fig. 5F,G). Autophagy is dependent on recruitment of ER membrane for autophagosome formation, and CHOP can induce cell death through both stimulation of apoptosis and inhibition of autophagy^42^. Accordingly, EIPA- and HMA treatment was associated with marked accumulation of LC3B-II and p62, the combination of which is indicative of arrested autophagy (Fig. 5F).

The Ser/Thr kinases Extracellular Signal-Regulated Kinase (ERK) and Akt are major survival kinases, which are regulated in many stress conditions, including ER stress and paraptosis. These kinases can contribute to both survival and death signalling, and are targets of clinical anticancer therapies^43,44^. MCF-7 cells were therefore subjected to treatment with EIPA with and without ERK and Akt inhibitors (Fig. 5H). ERK, but not Akt, activity was increased upon EIPA treatment, and both kinases were effectively inhibited by their inhibitors, U0126 and Akti, respectively. Strikingly, ERK inhibition strongly potentiated EIPA-induced CHOP upregulation and PARP cleavage (Fig. 5H). This suggests that ERK activation under these conditions may be a compensatory survival mechanism, inhibition of which synergizes with EIPA in eliciting MCF-7 cell death.

Together, these results indicate that the cytotoxic effects of pyrazinoylguanidine-type NHE1 inhibitors are multifactorial and include pronounced DNA damage, ER stress, mitochondrial fragmentation, autophagic arrest, and paraptosis.

## Discussion

The key novel findings of this work are that 5-substituted pyrazinoylguanidine-type NHE1 inhibitors accumulate in cancer cells grown under 3D conditions and exert potent, multifaceted cytotoxic effects unrelated to NHE1 inhibition. In marked contrast, the benzoylguanidine-based compounds, cariporide and eniporide, exert no detectable toxicity. To the best of our knowledge, this is the first study in which the effect of these inhibitors has been comprehensively studied across compounds and compared to CRISPR/Cas9-mediated ablation of NHE1. It is also the first in which this has been done in a 3D setting, which is superior to 2D models in predicting *in vivo* response to drug treatment^3,19,22^.

### Pyrazinoylguanidine cytotoxicity is NHE1-independent and cell-type specific

Genetic reduction or ablation of NHE1 reduces proliferation, invasiveness and *in vivo* growth of a wide range of cancer cells^5,6,8–10,33^. Small molecule NHE1 inhibitors have therefore been explored as an anticancer approach, alone or in combination treatment schemes^6,31,32,45^. However, evidence from 2D systems has shown that at high concentrations (20–40 µM, and up to 500 µM for amiloride, compare to IC_50_ values for NHE1 inhibition, Table 1), some pyrazinoylguanidine-type NHE1 inhibitors exert NHE1-independent cytotoxicity^24,25,28,46^. Furthermore, pyrazinoylguanidine-type inhibitors inhibit several other NHE isoforms than NHE1 and some additionally target the epithelial Na^+^ channel (ENaC) and Na^+^/Ca^2+^ exchangers^13–15^. In cancer cells, the long-term effects of pyrazinoylguanidine-type NHE1 inhibitors, which have generally been assumed to be downstream of NHE1 inhibition, include decreased proliferation, reduced metastatic potential and -viability, and sensitization to chemotherapy^32,45,47,48^. There are, however, reports of NHE1-unrelated effects of these compounds, including dysregulation of ER Ca^2+^ homeostasis^24^, reactive oxygen species production^25^, urokinase plasminogen activator inhibition^26^, and cell death^27,28^. The present study is the first to use NHE1 CRISPR/Cas9 KO in conjunction with extensive pharmacological analyses, to unequivocally establish the NHE1-independence of these effects. Interestingly our work mirrors recent reports taking advantage of CRISPR/Cas9 mutagenesis to demonstrate that the effect of many anticancer drugs in clinical trials is unrelated to their presumed target^29,30^.

Our first observation was that EIPA treatment lead to a dramatic loss of breast cancer spheroid viability, both as monotherapy and in combination with tamoxifen or chemotherapy. In contrast, the benzoylguanidine-type NHE1 inhibitor cariporide had no effect, in agreement with our previous observations in MCF-7 spheroids^33^. Both compounds were effective in inhibiting NHE1, albeit cariporide seemed less effective than EIPA in MDA-MB-231 cells grown in 3D. In contrast to MCF-7 cells, MDA-MB-231 cells exhibit strong V-ATPase relocalization to the plasma membrane upon adaptation to extracellular acidification (L.E.L., S.F.P, unpublished) – a condition encountered in the 3D environment. We therefore speculate that the incomplete inhibition of net pH_i_ recovery by cariporide in MDA-MB-231 spheroids may reflect a cariporide-insensitive contribution to pH_i_ regulation that underlies the incomplete inhibition in these spheroids. Critically, this does not affect the study conclusions, because the markedly different toxicity of EIPA vs cariporide was also seen in both MCF-7 spheroids and in 2D, where the two compounds had equal inhibitory effects on NHE1.

Unequivocally establishing the NHE1-independence, EIPA-induced cytotoxicity was indistinguishable between NHE1 KO and WT spheroids from several breast cancer types. EIPA-induced toxicity was also pronounced in several other cell types. While non-cancer cells were generally less sensitive than cancer cells, they were not fully unaffected and some cancer cells were also relatively insensitive to pyrazinoylguanidine-induced cytotoxicity. This warrants caution against previous proposals from 2D studies that these compounds are cancer cell-selective^46^ and shows that sensitivity must be determined on a cell type-to-cell type basis. In contrast, cariporide had no effect on spheroid viability for any of the cell lines tested, in agreement with previous reports^28,49^.

### Pyrazinoylguanidine cytotoxicity in 3D is associated with intracellular compound accumulation

The use of 3D models is essential to anticancer drug studies, because they mimic the tumour microenvironment^3,19,20^, including the dense extracellular matrix (ECM), tortuous extracellular space, and acidic pH, as low as 6.0 in the tumour core^9^. In our study, we employed 3D spheroids prepared both with and without exogenous addition of matrix components, reflecting the varying capacity of different cancer cells for cell-cell adhesion. Given the propensity of cancer cells for endogenous matrix production^50^, and the tight spheroid morphology, we expect all spheroids used to exhibit a tortuous extracellular space. However, also spheroids prepared without exogenous ECM addition are suitable for *in vitro* modelling of tumour drug response^19,22^. Under these conditions, weak base drugs accumulate in the extracellular space and in acidic organelles (which are also more acidic at acidic extracellular pH^51^) than in the less acidic cytosol and nucleus (the phenomenon of “ion trapping”). For chemotherapeutic drugs destined to function in the nucleus, this can lead to physiological treatment resistance, i.e. reduced drug concentration at the site of action^12,23^. The benzoylguanidine-type inhibitors cariporide and eniporide are weak acids with pK_a_ values of 6.2 and 6.0, respectively^35,36^. Based on their chemical properties, they are thus expected to preferentially partition into the cytosol, whereas the pyrazinoylguanidines, which are weak bases (pK_a_ values 8.5–8.7^15,34^), should partition into acidic compartments. In congruence with this, EIPA and HMA, which were profoundly cytotoxic, accumulated most markedly in perinuclear lysosomes.

Dose-response analysis showed that cariporide and eniporide induced no detectable cytotoxicity even at the highest doses tested. The pyrazinoylguanidines exhibited a relative toxicity largely correlating with their efficacy in inhibiting NHE1 (EIPA ≈ HMA > DMA > amiloride, Table 1), despite the fact that we unequivocally showed that their cytotoxicity is NHE1-independent. Thus, the order of potency in inhibiting growth was HMA > EIPA > DMA > amiloride in MCF-7 spheroids, and HMA > EIPA > amiloride> DMA in MDA-MB-231 spheroids. This sequence however also correlates well with measured or expected lipophilicity^13,15^, and hence partitioning into membranes and thus permeability (the more hydrophobic the substituted groups, the greater expected lipophilicity, thus HMA should be the most lipophilic of the tested pyrazinoylguanidines, Table 1).

The pyrazinoylguanidines studied in this work bear a similar architecture comprising a guanidine group connected to a flat aromatic ring by an amide bond. However, they also have noticeable differences. In contrast to cariporide and eniporide, which are built on a phenyl aromatic ring, amiloride, DMA, EIPA and HMA are built on a heterocyclic pyrazine ring. This structure bears two nitrogen atoms at positions 1 and 4, whose electronegativity influence the electron distribution, making this ring slightly electron deficient. Furthermore, these Lewis basic N lone electron pairs render pyrazine weakly basic. The pyrazinoylguanidine compounds tested in this study all feature a 6-Cl halogen atom, meta to the 2-acyl guanidine on the pyrazine ring. While the Cl is more electronegative than C, the overall consequence of the substitution of C-H for C-Cl produces a net increase in the hydrophobicity of the molecule (see^15^). Cariporide and eniporide bear a sulfonyl group at the same position that has very strong electron withdrawing properties giving these molecules, which bear a large hydrophobic component (see below), amphiphilic properties.

Both classes of molecules have groups in para position to the acylguanidine moiety. These are a single amino group (amiloride), and hydrophobic 5-*N* alkyl or cycloalkyl moieties (DMA, EIPA, HMA), isopropyl (cariporide) or a pyrrole group (eniporide). Lastly, pyrazinoylguanidines bear an ortho 3-amino group that, similar to the 5-amino group, is likely to affect the pKa value of the guanidine group by resonance. This group is not present in cariporide and is replaced by a methyl in eniporide. Taken together, the cellular toxicities of amiloride, DMA, EIPA and HMA observed here correlate well with the size and hydrophobicity of their 5-*N* substituents (see also Table 1). It is tempting to propose that these hydrophobic substituents affect the overall ability of these molecules to partition into membranes, penetrate cells and accumulate in intracellular compartments. Furthermore, compared to cariporide and eniporide, the structures of amiloride derivatives feature a number of potentially reactive basic groups. This makes these molecules more likely to interact and react with intracellular components. Beyond these observations, the numerous differences between these two classes of molecules, as listed above, make it impossible to unequivocally assign the observed toxicity to a single chemical group. Thus, future studies should narrow down the precise functional group(s) responsible for the toxic effects and equally important assess the potential NHE1-independent effects of bi- or tricyclic NHE1 inhibitors such as zoniporide, SM-20550, and KB-R9032.

This work shows that the potent NHE1-independent cytotoxic effects of EIPA and HMA in long-term 3D culture occur below or at the concentration range generally applied for NHE1 inhibition (i.e. at concentrations as low as 1 µM, compared to the 5–10 µM widely used for NHE1 inhibition). This effect involves accumulation in the endo-/lysosomal compartment and is likely to be exacerbated by the increased size and acidity of this compartment under the acidic extracellular conditions of tumours, as mimicked in spheroids^51^. Our findings also provide an explanation for the apparently paradoxical finding that the cytotoxicity of pyrazinoylguanidine-type inhibitors is greatest under acidic conditions^52^. While these compounds have not yet been used in clinical trials, it seems highly probable that a similar time-dependent accumulation would be seen upon long-term treatment of cancer patients. In conjunction with the tendency of the cytotoxic effect to be more profound in cancer cells than in normal cells, this makes the pyrazinoylguanidines potentially interesting in anticancer therapy, as discussed further below.

### Pyrazinoylguanidines elicit profound ER stress, reduced autophagic flux, and paraptosis

The pattern of accumulation indicated that the pyrazinoylguanidine compounds exert their cytotoxic effects at least in part by disrupting lysosome- and ER function. Consistent with this notion, EIPA and HMA treatment caused profound upregulation of the ER stress-induced transcription factor CHOP and increased expression of both LC3B-I and –II and p62. The accumulation of both lipidated LC3B-II and of p62 is indicative of stalled autophagic flux, as expected if lysosomal function is disrupted. NHE6, −7, and 9 are present in the endo-lysosomal pathway, however, a role for inhibition of these isoforms seems unlikely to explain our findings as, similar to NHE1, these transporters are sensitive to both benzoyl- and pyrazinoylguanidines^13^. The same is true for NHE2, which is the only other plasma membrane NHE expressed at non-negligible levels across most cell types studied here (Human Protein Atlas, mRNA levels). The upregulation of CHOP is consistent with an earlier report that EIPA and HMA elicit ER Ca^2+^ depletion in endothelial cells in 2D culture^24^. The reduction of autophagic flux is likely secondary to both ER stress^42^ and lysosomal dysfunction, precluding autophagosome formation. Consistent with the former, our 2D experiments revealed induction of massive perinuclear vesicles surrounded by mitochondrial- and ER markers. This pattern is characteristic of paraptosis – a form of caspase-independent cell death associated with perinuclear swelling of ER and mitochondria^39,40^. Finally, in congruence with the reported EIPA- and HMA-induced ER Ca^2+^ depletion^24^, paraptosis is dependent on ER- and mitochondrial Ca^2+^ dysregulation in a manner proposed to involve Ca^2+^ flux from ER to mitochondria at ER-mitochondrial contact sites^40^. EIPA and HMA treatment also caused γH2AX upregulation, congruent with the notion that some pyrazinoylguanidines (such as amiloride and EIPA) can intercalate into DNA^41^. However, given the lack of p53 upregulation in the highly sensitive MCF-7 cells (which are p53-wild type), and the reported resistance of MCF-7 cells to DNA-damage-induced apoptosis^53^, it seems unlikely that DNA damage contributes substantially to pyrazinoylguanidine-induced death, at least in MCF-7 cells.

### Perspectives and relevance to basic research and anticancer treatment

This work clearly establishes that effects of pyrazinoylguanidine NHE inhibitors cannot be taken as evidence of a role of NHEs, warranting caution in their use and meaning that the existing literature using these compounds should be re-evaluated. It remains clear, however, from studies using other tools, that NHE1 is important for the growth and development of many cancers^5,6,8–10,33^. Consistent with previous reports^6,32,54^, our results point to an additive effect of EIPA in combination with tamoxifen, combination chemotherapy and ERK inhibition. In our hands, EIPA-induced cytotoxicity was particularly strongly potentiated by inhibition of ERK, a highly interesting target in e.g. KRAS mutated cancers^43,55^. This suggests a substantial potential for combination therapy which should be investigated in future studies. Specific questions to be explored include effects of sub-lethal doses of pyrazinoylguanidine-type NHE1 inhibitors, combination with other anticancer treatments, and staggered treatment combinations, taking into account the potentially different latency of action of EIPA and other treatments tested.

An important implication of these findings is that in NHE1-dependent cancers, pyrazinoylguanidine-type NHE1 inhibitors are likely to exert dual, NHE1-dependent and -independent anticancer effects, including ER stress and paraptosis. Paraptosis is attractive as an anticancer death pathway because it is independent of pro-apoptotic Bcl-2 proteins and effector caspases, which are frequently downregulated in cancer cells. In addition to exploring combination treatments, future work should establish whether the cytotoxicity is sufficiently cancer specific to be useful in a therapeutic context and identify cancer types likely to benefit from this treatment. Interesting possibilities to explore would include KRAS- or ERK-driven tumours with high NHE1 expression.

*In conclusion*, we show that pyrazinoylguanidine-type NHE1 inhibitors exert potent, cell type-dependent, NHE1-independent cytotoxic effects on cancer cells grown as 3D spheroids, at concentrations generally assumed to specifically inhibit NHE1. We propose that the cytotoxicity of the pyrazinoylguanidines reflects their hydrophobicity in conjunction with weak base properties, collectively resulting in their trapping and accumulation in acidic organelles, leading to DNA damage, ER stress, paraptosis, and stalling of autophagic flux. We suggest that both the NHE1-dependent and –independent effects of these compounds may render them attractive in the context of anticancer therapy.

## Methods

A table describing relevant equipment and settings is provided as Supplementary Table S1.

### Antibodies and reagents

A table containing all employed antibodies as well as their cellular target, supplier and catalogue number is provided as Supplementary Table S2.

Cariporide was a gift from Sanofi-Aventis and 5-(*N*-ethyl-*N*-isopropyl) amiloride (EIPA) (#E3111) was from Thermo-Fisher. β-actin antibody (#A5441), tamoxifen (#T5648), cisplatin (#P4394), 5-fluorouracil (5-FU) (#F6627), 5-(*N*,*N*-Dimethyl) amiloride (DMA) (#A4562), 5-(*N*,*N*-Hexamethylene) amiloride (HMA) (#A9561) and amiloride (#A7410) were from Sigma-Aldrich, and doxorubicin (#120629) and eniporide (#HY-106150B) from Abcam and MedChemExpress, respectively. Akt (Akti, #124018) and MEK (U0126, #662005) inhibitors were from Calbiochem. Horseradish Peroxidase (HRP)-conjugated anti-mouse- (#P0447) and anti-rabbit (#P0448) antibodies were from Dako, and Alexa Fluor anti-mouse (#A11019) and anti-rabbit (#A11070) fluorophore-conjugated secondary antibodies were from Invitrogen.

### Cell lines and cell culture

MDA-MB-231 (#HTB-26, from ATCC) and MCF-7 S9 cells (a gift from Dr. L. Rønnov-Jessen, University of Copenhagen) and their respective CRISPR/Cas9-mediated NHE1 KO variants^5,33^ were cultured in DMEM1885 (University of Copenhagen, Panum 22-2-24, #015), supplemented with 6% and 10% fetal bovine serum (FBS, Sigma-Aldrich, F9665) for MCF-7 and MDA-MB-231 WT and KO cell lines respectively, 1% Penicillin/Streptomycin (P/S, Sigma-Aldrich, #P0781) and 1% MEM Non-Essential Amino Acids 100× (Gibco, #11140-035). MCF10A cells were cultured in DMEM (Gibco, #41966) mixed 1:1 with F12 (Sigma-Aldrich, #N6658) plus 5% heat-inactivated Horse serum, 1% P/S, 20 ng/mL EGF (Sigma-Aldrich, #E9644), 0.5 µg/mL Hydrocortisone (H0888, Sigma-Aldrich) and 1% Insulin (Gibco, #41400045). BxPC-3 and T47D cells were grown in RPMI (Gibco, #61870-010), while HCT116, SKBr-3, and NIH3T3 cells were cultured in DMEM (Gibco, #31885-023, #41966-029 and #32430-027 respectively) supplemented with 1% P/S and 10% FBS. Medium for T47D cells was further supplemented with 0.5% Insulin-Transferrin-Selenium (Gibco, #41400-045). Cells were cultured at 37 °C, 5% CO_2_, passaged at a confluence of ~80% and discarded after passage 23.

### Spheroid growth assay

1–2 × 10^3^ cells were seeded per well in round-bottomed, ultra-low attachment (ULA) 96-well plates (VWR, #4441020) in 200 µL of their respective media and grown for the indicated number of days (7–9) at 37 °C. Media for MDA-MB-231, MCF10A and SKBr-3 cells were supplemented with 1.5% Geltrex LDEV-Free Reduced Growth Factor Basement Membrane Matrix (Thermo-Fisher, #A1413202) and the plates centrifuged for 15 min at 750 RCF immediately after seeding. Where indicated, cariporide (10 µM), eniporide (2–20 µM), amiloride (10–500 µM), EIPA (0–10 µM), HMA (5–40 µM), or DMA (5–80 µM) were added after 2 days of spheroid growth, either alone or simultaneously with anti-cancer treatments: Tamoxifen (0.5, 1, or 2 µM), or the combination Cisplatin (18.75 nM) plus Doxorubicin (18.75 nM) plus 5-FU (0.0625 nM). Drug concentrations were determined based on pilot dose-response experiments (Supplementary Fig. S1), and chosen to elicit an intermediate level of cell death under the conditions tested. Every 2–3 days, 100 µL medium (incl. inhibitors or anti-cancer drugs) was replaced and light microscopic images (Leica MZ16 microscope, Germany) of the spheroids were acquired. Treatment was terminated at day 9 of growth (day 7 of drug treatment), except for the MDA-MB-231 cells in the experiments in Fig. 1, which were terminated at day 7.

### Spheroid-based cell viability assays

100 µL medium was replaced with 50 µL CellTiter-Glo 3D Reagent (Promega, #G9683) on day 7 or 9. The plates were shaken for 5 min, incubated for 25 min at room temperature (RT) and the luminescent signal was recorded using FLUOStar Optima (BMG). Data was analysed using Excel and Graphpad Prism.

EC_50_ values were determined in Graphpad Prism by nonlinear regression using the built-in ‘Dose-response curves -Inhibition’ model: *Y* = *bottom* + *(Top-bottom)/(1* + *10^((X-LogIC50))*, where X is the logarithm of the inhibitor concentration, bottom is the minimum and top the maximal response, and Y is the cell viability response at the corresponding inhibitor concentration. The reported 95% confidence intervals (CI) of the EC_50_ are depicted in Table 1. For MDA-MB-231 spheroids, the EC_50_ for EIPA was determined in Excel using the slope of linear regression, as the inhibitor concentrations employed were not high enough to cause a sufficient minimum response to fit this model.

### Measurement of intracellular pH

MDA-MB-231 and MCF-7 spheroids grown for 4 days were transferred to poly-L-lysine-coated (0.01%, Sigma, #P4707) dishes (WillCo Wells #HBST-3522), and incubated for 30 min at 37 °C. Spheroids were loaded with 3.2 µM BCECF-AM, incubated for 30 min at 37 °C, washed in HCO_3_^−^ containing Ringer (in mM, 118 NaCl, 25 NaHCO_3_, 5 KCl, 1 MgSO_4_, 1 Na_2_HPO_4_, 1 CaCl_2_, 3.3 3-(N-morpholino)-propanesulfonic acid (MOPS), 3.3 2-[Tris(hydroxymethyl)-methylamino]-ethanesulfonic acid (TES), 5 HEPES, adjusted with NaOH to pH 7.4) heated to 37 °C and placed in a 37 °C with 5% CO_2_/air chamber with solute perfusion on a Nikon Eclipse T*i* microscope. pH_i_ was recorded in real time essentially as in^5^. Briefly, BCECF fluorescence was measured ratiometrically at defined regions of interest (ROIs) by alternating excitation between 440 and 485 nm and recording emission at 520 nm. Steady state pH_i_ was measured for 5 min in HCO_3_^−^-Ringer pH 7.4, followed by a ∼10 min 20 mM NH_4_Cl pre-pulse resulting in acidification upon its washout with HCO_3_^−^-Ringer pH 7.4. Recovery was recorded for 10 min and the recovery rate determined as the slope of the first 2 min of the recovery curve.

### Immunoblotting

*Lysis of cells in 2D culture*. Cells were grown to 70–90% confluency in Petri dishes, washed in ice-cold PBS and lysed (Lysis buffer (LB): 1% SDS, 10 mM Tris-HCl, 2 Complete mini protease inhibitor tablets (#11836153001, Roche Diagnostics GmBH) and 1 mM NaVO_3_, pH 7.5, heated to 95 °C). Lysates were homogenized by sonication and centrifuged for 5 min at 20,000 g at 4 °C to remove cell debris.

*Lysis of cells in 3D culture*. Spheroids were gently collected in Eppendorf tubes, followed by one wash in ice-cold PBS and lysis in LB for 5–10 min at RT with intermittent vigorous vortexing. Sonication, homogenization and centrifugation were as described for 2D culture.

### SDS-PAGE and immunoblotting of 2D and 3D cultures

Lysate protein content was determined using the Bio-Rad DC Protein Assay kit (Bio-Rad Laboratories, #500-0113 (Working reagent A); #500-0114 (Working reagent B); #500-0115 (Working reagent S)). Sample protein contents were equalized with ddH_2_O, and NuPAGE LDS 4x Sample Buffer (Invitrogen, #NP0007 + 0.5 M Dithiothreitol (DTT) was added. Proteins were separated by SDS-PAGE using Criterion TGX Precast Gels, Tris/Glycine/SDS running buffer (Bio-Rad, #161 0732) and Benchmark ladder (Invitrogen, #10747-012) and transferred to Trans-blot Turbo 0.2 µm nitrocellulose membranes (Bio-Rad, #170-4159) using the Trans-blot Turbo Transfer System (Bio-Rad, #10022518). After staining with Ponceau S (Sigma-Aldrich, #P7170-1L), membranes were blocked (blocking buffer: 5% nonfat dry milk in TBST (0.01 M Tris/HCl, 0.15 M NaCl, 0.1% Tween 20)) for 1 h at 37 °C. Subsequently, they were incubated overnight at 4 °C with primary antibodies, washed in TBST, and incubated with HRP-conjugated secondary antibodies for 1 h, RT. Membranes were washed in TBST and developed using Pierce ECL Western blotting substrate (Thermo Scientific, #32106), on a Fusion FX developer (Vilber Lourmat). Band intensity was quantified using ImageJ software.

### Measurements of inhibitor accumulation

#### Monolayer cells

24 h after seeding on glass coverslips, cells were treated with cariporide, EIPA or HMA for 2 days before fixation in 2% paraformaldehyde (PFA, Sigma, #47608) and visualization employing Olympus IX83 microscope with a Yokogawa scanning unit, using a 60X/1.4 NA objective and aquisition using CellSens Dimension software. Intensity adjustment was performed using ImageJ software.

#### Spheroids

Cells were seeded for spheroid formation either 2 or 7 days prior to measurement of accumulation and treated with cariporide, EIPA or HMA for 4 h or 5 days, respectively. Fluorescence intensity of the inhibitors was measured on a Nikon Eclipse T*i* microscope employing EasyRatioPro software (PTI) at wavelengths 340–400 nm with 5 nm band intervals. Emission was recorded at 420 nm and data was analysed in Excel and Graphpad Prism.

### Immunofluorescence analysis

Cells cultured on glass coverslips were treated with cariporide or EIPA for 2 or 4 days. Cells were washed in cold PBS, fixed in either 2% PFA for 15 min at RT and 30 min on ice or 4% PFA for 30 min at RT and washed twice in TBST. Cells fixed in 2% PFA were permeabilized in 0.5% Triton-X-100 (Plusone, #17-1315-01) in TBST and blocked using 5% Bovine Serum Albumin (BSA, Sigma-Aldrich, #A7906). Cells fixed in 4% PFA were quenched in 50 mM NH_4_Cl in TBST and permeabilized and blocked in 5% BSA with 0.1% Saponin (Sigma-Aldrich, #47036). The cells were incubated with primary antibodies diluted in TBST plus 1% BSA overnight at 4 °C or for 1.5 h, RT, and with secondary antibodies, also in TBST plus 1% BSA, at RT for 1 h, followed by a 5 min DAPI (1:1000) incubation, wash in 1% BSA, and mounting in 2% N-propyl-gallate mounting medium (Sigma-Aldrich, #P3130). Fluorescently labeled proteins were visualized employing an Olympus IX83 microscope with a Yokogawa scanning unit, using a 60X/1.4 NA oil immersion objective and CellSens Dimension software. No image processing except merges, zooms, and color balance was performed. Image overlays and adjustments of the intensities were performed using ImageJ software.

### Inhibition of Akt and MEK

Cells were seeded in 6-well plates for 24 h prior to 48 h-treatment with EIPA (10 µM), Akti (1 µM), U0126 (10 µM) or a combination thereof. Lysates were prepared as described in the above section on immunoblotting.

### Propidium Iodide (PI)-staining of spheroids

On day 9, 100 µL medium was removed, spheroids were washed three times with heated PBS, 100 µL PBS containing 4 µM PI was added per well (2 µM final concentration) and plates were incubated at 37 °C for 10–15 min protected from light. Spheroids were washed three times in heated PBS and visualized employing an Olympus IX83 microscope with a Yokogawa scanning unit, using a 10X air objective. Subsequent image adjustments were performed using ImageJ software.

### Statistical analysis

Data are shown as representative images or as means with standard error of the mean (SEM) error bars as indicated. After verifying normal distribution of the data, one-way analysis of variance (ANOVA) followed by Tukey’s or Dunnett’s multiple comparisons post-test was used to test for significant differences when more than two groups were compared, while a paired or unpaired Student’s *t*-test, as relevant, was employed when only two groups were analysed.

## Supplementary information

Supplementary Information.

## Data Availability

The datasets used and/or analysed during the current study are available from the corresponding author on reasonable request.
