# Supplementary material for: Pyrazine ring-based Na+/H+ exchanger (NHE) inhibitors potently inhibit cancer cell growth in 3D culture, independent of NHE1
Source: Sci Rep. 2020 Apr 2;10:5800. doi: 10.1038/s41598-020-62430-z (PMC7118118; doi:10.1038/s41598-020-62430-z)
Supplement: Supplementary file 1 — Supplementary Information. [file 41598_2020_62430_MOESM1_ESM.pdf]

Pyrazine ring-based Na^+/H^+ exchanger (NHE) inhibitors potently inhibit cancer cell growth in 3D culture, independent of NHE1

Michala G. Rolver[1], Line O. Elingaard-Larsen[1], Anne P. Andersen[2],

Laurent Counillon[3], Stine F. Pedersen[1,]*

[1]Section for Cell Biology and Physiology, Department of Biology, Faculty of Science, University of Copenhagen, Copenhagen, Denmark;
[2]Centre for Medical Parasitology, Department of Immunology and Microbiology, Faculty of Health and Medical Sciences, University of Copenhagen, Copenhagen, Denmark.
[3]Université Côte d'Azur, CNRS, France LP2M, 28 Avenue de Valombrose, Nice.

Supplementary files

Supplementary file 1

Title of data:

Supplementary methods

Description of data:

Detailed descriptions of methods only used in the supplementary figures.

Measurement of intracellular pH in 2D

MDA-MB-231 and MCF-7 WT cell lines were seeded in 24-well plates 36 h prior to the experiment, then loaded with 2’,7’-bis-(2-carboxyethyl)-5-(and-6)-carboxyfluorescein acetoxymethylester (BCECF-AM, 1.6 or 3.2 µM) for 30 min at 37°C. Cells were washed twice in HEPES-buffered Ringer solution (in mM, 130 NaCl, 3 KCl, 20 HEPES, 1 $MgCl_2$, 0.5 $CaCl_2$, 10 NaOH pH 7.4), re-bathed in Ringer and placed in a FluoStar Optima plate reader thermostatted at 37°C. Emission was measured at 520 nm and excitation at 485 nm. After a baseline measurement (10 min) in Ringer, acidification was induced using the NH_4Cl pre-pulse technique: cells were exposed to 20 mM NH_4Cl for 5 min followed by 90 s in a Na^+-free Ringer (in mM, 3 KCl, 20 HEPES, 1 $MgCl_2$, 0.5 $CaCl_2$, 135 NMDGCl, pH 7.4). Na^+-containing Ringer was reintroduced, and pH_i recovery monitored, under control conditions or in presence of 10 µM cariporide or EIPA (10 min). In each experiment, calibration of BCECF fluorescence to pH_i was performed with the high K^+/nigericin method [40] (High KCl-Ringer in mM: 140 K^+, 10 HEPES, 1 MgCl, 0.5 $CaCl_2$. 50 µM nigericin added individually to each well). Normalization between experiments was performed by setting the fluorescence measured in high KCl-Ringer pH 7.4 to 1. The recovery rate was calculated as the slope of the initial linear part of the recovery curve.

Lentiviral knockdown of NHE1 in MCF-7 cell line

Lentiviral knockdown (KD) of NHE1 in MCF-7 cells was performed as described in [38].

Supplementary Figure S1

Title of data:

Dose-response experiments for determination of anti-cancer therapy doses

Description of data:

A) MCF-7 spheroids were grown for 9 days and treated with cariporide (10 µM), tamoxifen (0.5, 1, or 2 µM) or a combination thereof as indicated, on day 2, 4 and 7. DMSO served as vehicle for tamoxifen. A cell viability assay was performed on day 9. Error bars denote SEM. 3 n. B) MDA-MB-231 spheroids were grown for 7 days and treated with decreasing concentrations of chemotherapy (cisplatin (0.3-0.01875 µM), doxorubicin (0.3-0.01875 µM) and 5-FU (2-0.0625 nM)) on day 2 and 4. Cell viability was assessed on day 7. Error bars denote SEM. 2 n. C) Scheme of the chemotherapeutic doses corresponding to the colors in B.

A

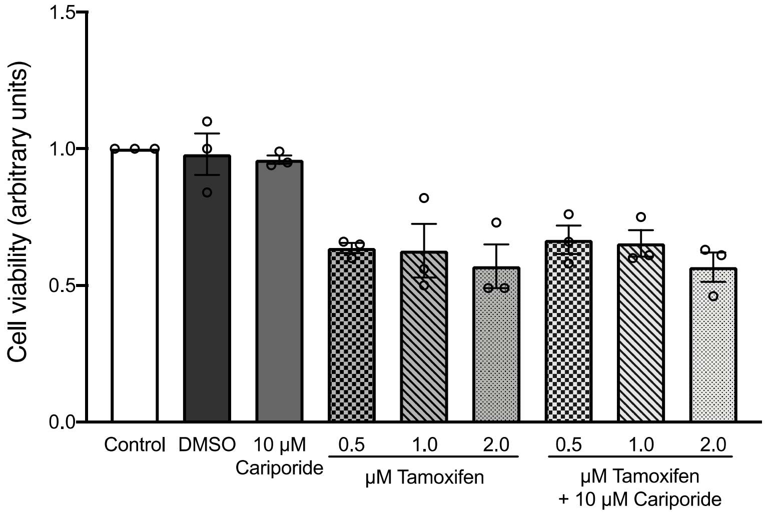

B

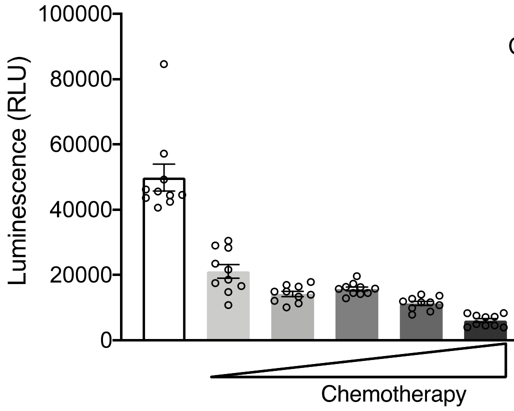

C

	Cisplatin (nM)	Doxorubicin (nM)	5-FU (nM)
	18.75	18.75	0.0625
	37.5	37.5	0.125
	75.0	75.0	0.5
	150	150	1
	300	300	2

Supplementary Figure S2

Title of data:

Prominent cell death throughout MCF-7 spheroids upon EIPA treatment.

Description of data:

MCF-7 cells were grown as spheroids for 9 days and treated with cariporide (10 µM), EIPA (10 µM) or Tamoxifen (2 µM) either as monotherapy or as a combination thereof on day 2, 4 and 7. Light microscopic images of spheroid growth were acquired on day 2, 4, 7 and 9. PI staining was performed on day 9. Images are representative of 3 independent experiments. Scale bar: 100 µm.

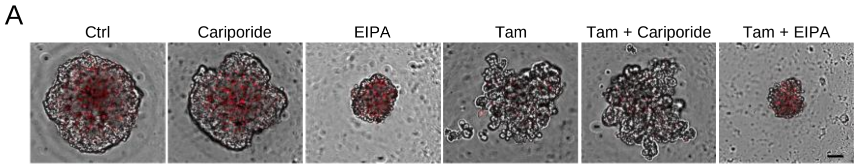

Supplementary Figure S3

Title of data:

Both cariporide and EIPA significantly delay the pH_i recovery rate in MCF-7 and MDA-MB-231 cells.

Description of data:

MCF-7 and MDA-MB-231 cells were loaded with BCECF-AM and acidified using the NH_4Cl pre-pulse technique. (NH_4Cl: 20 mM, 5 min). pH_i recovery was monitored spectrofluorimetrically in presence or absence of cariporide (10 µM) or EIPA (10 µM). Experiments were performed under HCO_3^--free conditions to eliminate contributions from HCO_3^--dependent transporters. A+C) Representative traces demonstrating BCECF-fluorescence as a function of time during pH_i recovery of MCF-7 (A) and MDA-MB-231 (C) cells after NH_4Cl pre-pulse. Fluorescence was normalized to that in high KCl/nigericin pH 7.4. Representative of 3-4 independent experiments. B+D) Quantification of pH_i recovery rate measured for MCF-7 (B) and MDA-MB-231 (D) cells as the slope of a linear regression line fitted to the first 84 s of recovery. Error bars denote SEM. 3-4 n. Statistical significance determined using a one-way ANOVA test with Tukey's multiple comparisons post-test. In panel A, the *p*-value is 0.0062 and 0.0063 for control compared to cariporide and EIPA, respectively, while in panel B, the *p*-value is 0.0154 and 0.0180 for control compared to cariporide and EIPA, respectively.

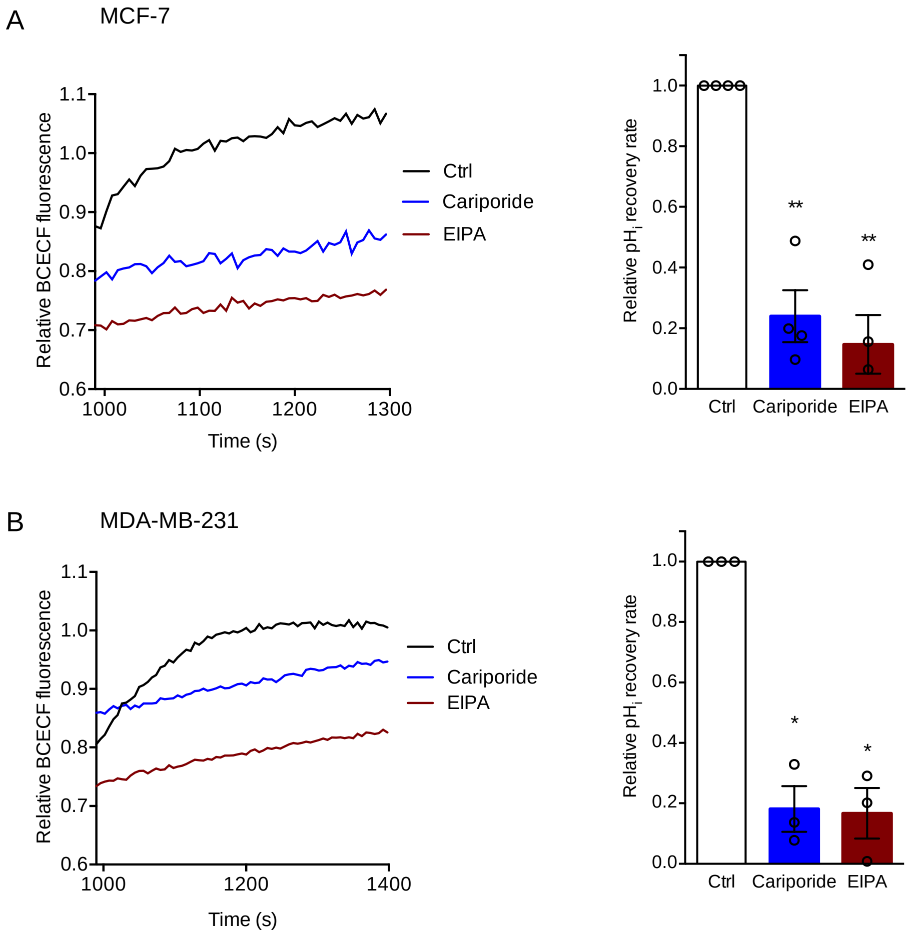
A
MCF-7
Relative BCECF fluorescence
Time (s)
Ctrl
Cariporide
EIPA
Relative pH$_i$ recovery rate
**
**
B
MDA-MB-231
Relative BCECF fluorescence
Time (s)
Ctrl
Cariporide
EIPA
Relative pH$_i$ recovery rate
*
*

Supplementary Figure S4

Title of data:

Cariporide does not affect the cell viability of cancer- and non-cancer cell lines

Description of data:

Spheroids of the human cancer cell lines HCT116, BxPC-3, T47D, SKBr-3, MDA-MB-231 and MCF-7 (colon, pancreatic and 4 different breast cancer subtypes, respectively) and the murine fibroblast cell line, NIH3T3, were grown as spheroids for 9 days and treated with cariporide (10µM) on day 2, 4, and 7. A) Bar plot displaying cell viability at 10 µM cariporide on day 9 for each cell line. Error bars denote SEM. 3-6 n. Dotted grey line represents control spheroids. Values from Figure 1B and D are included for MCF-7 and MDA-MB-231.

A

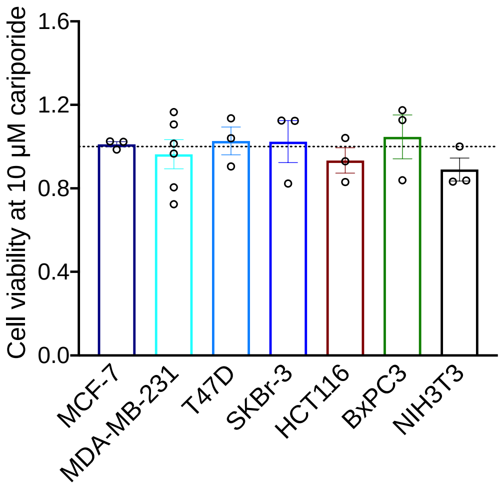

Supplementary Figure S5

Title of data:

EIPA, but not cariporide, potently reduces viability of MCF-7 NHE1 KD spheroids.

Description of data:

Stable NHE1 KD MCF-7 cells were grown for 9 days and treated with cariporide (10 µM), EIPA (10 µM) or tamoxifen (2 µM) either as monotherapy or as a combination thereof on day 2, 4 and 7. DMSO served as vehicle for tamoxifen. Light microscopic images of spheroid growth were acquired on day 2, 4, 7 and 9. Cell viability assay was performed on day 9. A) Representative images of MCF-7 spheroids. 3 n. Scale bar: 100 µm. B) Day 9 viability of MCF-7 spheroids. One-way ANOVA test with Tukey's multiple comparisons post-test was used to determine statistically significant differences between treatment groups. * and # denote significant differences between the treatment condition relative to DMSO and between two treatment conditions, respectively. The *p*-value is < 0.0001, 0.0455, < 0.0001, 0.0032 and 0.0336 for control compared to EIPA, tamoxifen, tamoxifen + cariporide, tamoxifen + EIPA and tamoxifen compared to tamoxifen + EIPA, respectively. Error bars denote SEM. 3 n.

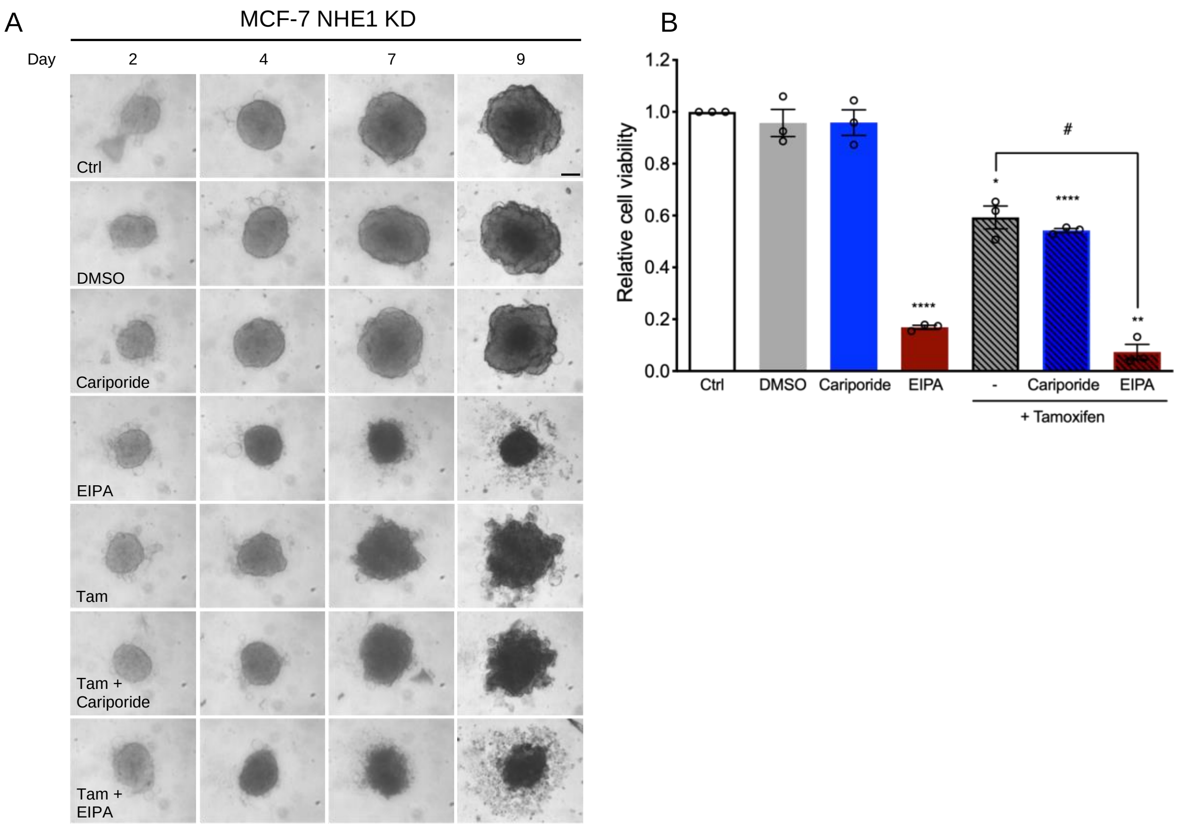

A
MCF-7 NHE1 KD
Day
2
4
7
9
Ctrl
DMSO
Cariporide
EIPA
Tam
Tam +
Cariporide
Tam +
EIPA
B
Relative cell viability
1.2
1.0
0.8
0.6
0.4
0.2
0.0
#
*
****
****
**
Ctrl
DMSO
Cariporide
EIPA
-
Cariporide
EIPA
+ Tamoxifen

Supplementary file: Figure S6

Title of data:

Full-length versions of western blots

Description of data:

Original, full-length and unprocessed version of all western blots shown throughout the paper.

Fig 3A

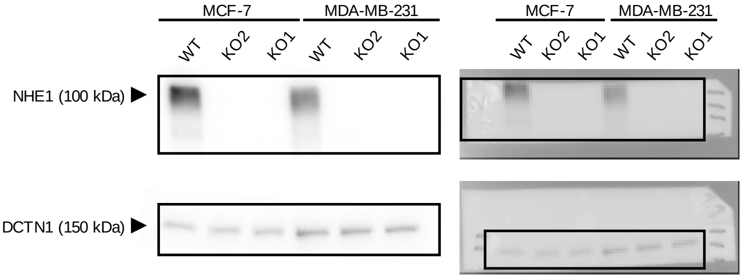

Fig 5A

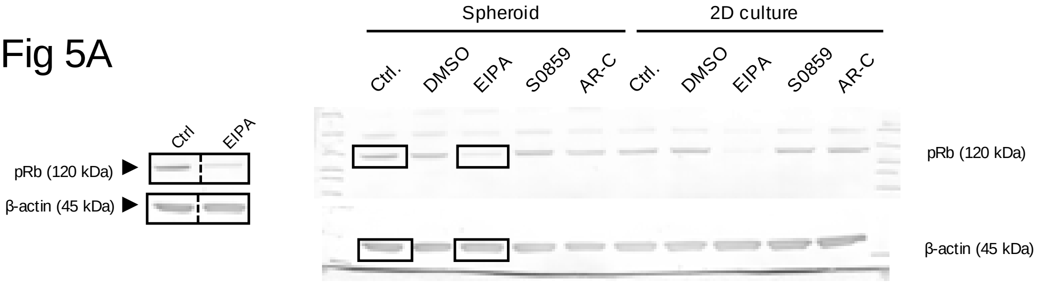

Fig 5B

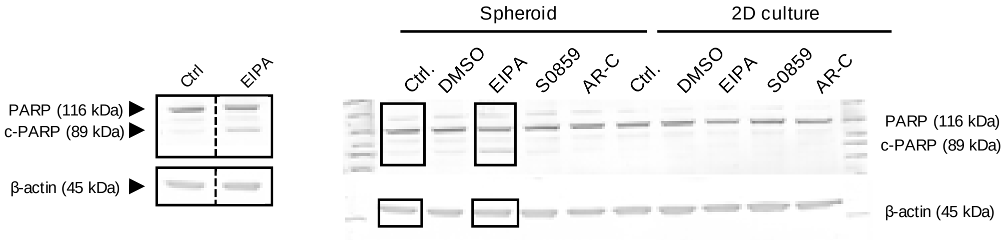

Fig 5C

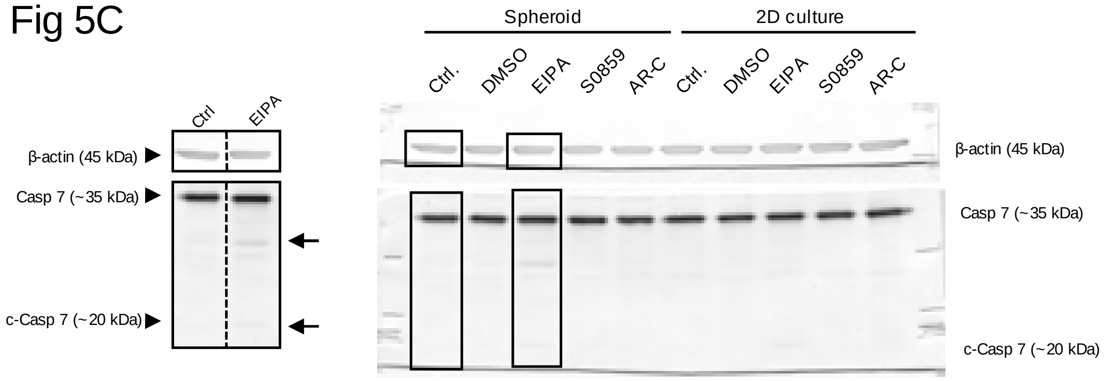

Fig 5D

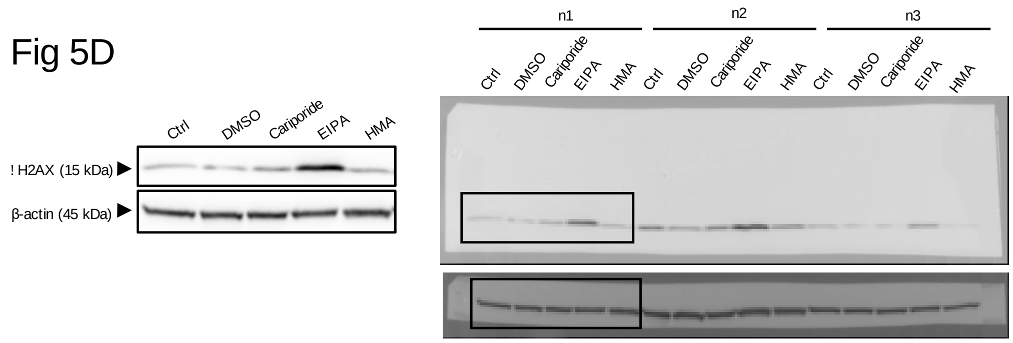

n1
n2
n3
Ctrl
DMSO
Cariporide
EIPA
HMA
! H2AX (15 kDa)
β-actin (45 kDa)

Fig 5F

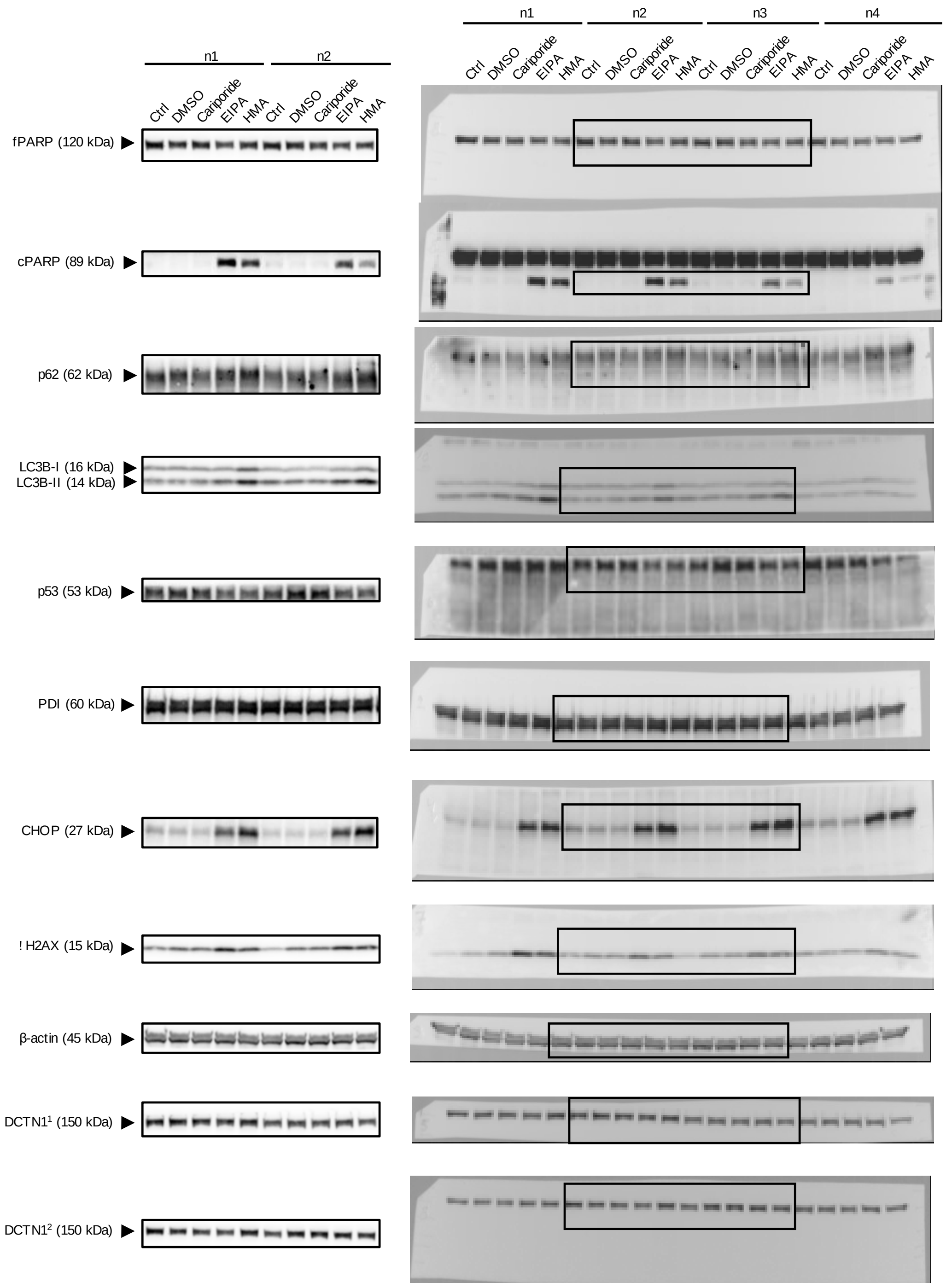

n1
n2
n3
n4
Ctrl
DMSO
Cariporide
EIPA
HMA
fPARP (120 kDa)
cPARP (89 kDa)
p62 (62 kDa)
LC3B-I (16 kDa)
LC3B-II (14 kDa)
p53 (53 kDa)
PDI (60 kDa)
CHOP (27 kDa)
! H2AX (15 kDa)
β-actin (45 kDa)
DCTN1¹ (150 kDa)
DCTN1² (150 kDa)

Fig 5H

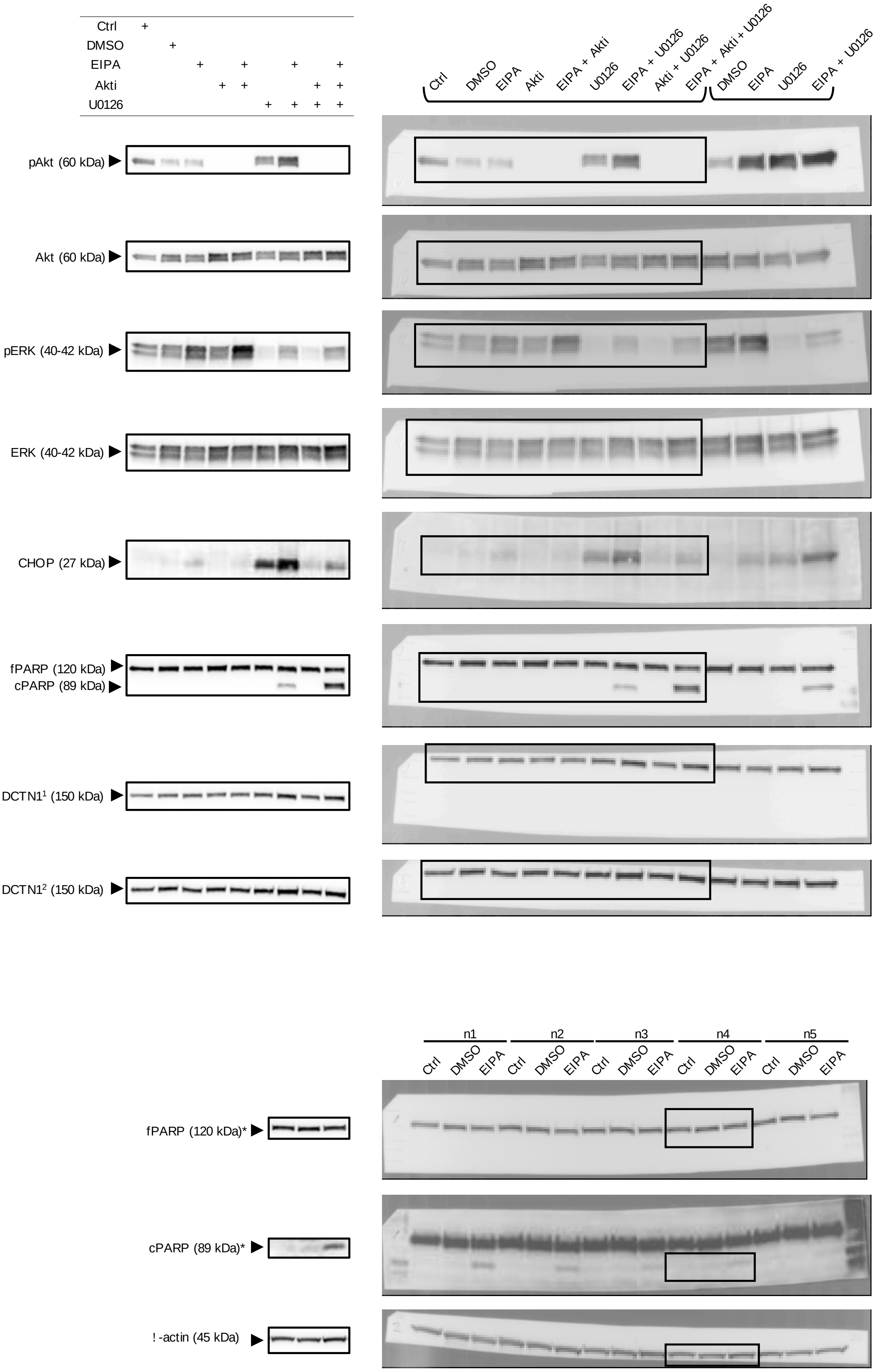
Ctrl
DMSO
EIPA
Akti
U0126
pAkt (60 kDa)
Akt (60 kDa)
pERK (40-42 kDa)
ERK (40-42 kDa)
CHOP (27 kDa)
fPARP (120 kDa)
cPARP (89 kDa)
DCTN1¹ (150 kDa)
DCTN1² (150 kDa)
EIPA + Akti
EIPA + U0126
Akti + U0126
EIPA + Akti + U0126
EIPA + U0126
n1
n2
n3
n4
n5
fPARP (120 kDa)*
cPARP (89 kDa)*
! -actin (45 kDa)

Supplementary file: Table S1

Title of data:

Equipment and settings table

Description of data:

A table containing details on the equipment and settings used for acquiring and processing the images shown throughout the paper.

All images were acquired at RT except for the intracellular pH measurements which were performed at 37 C.

Figure number	Instrument	Settings	Image processing software	Processing	Pixels	DPI	Comments
1A	Leica MZ16 light microscope (Germany)	10X magnification	Powerpoint	Cropping + grey-scale	1280x1024 pixels	72 pixels/inch	
1C	Nikon light microscope (Japan)	10X magnification	ImageJ and powerpoint	Cropping + grey-scale	2560x1920 pixels	72 pixels/inch	
2C	Leica MZ16 light microscope (Germany)	10X magnification	Powerpoint	Cropping + grey-scale	1280x1024 pixels	72 pixels/inch	
3B	Leica MZ16 light microscope (Germany)	10X magnification	Powerpoint	Cropping + grey-scale	1280x1024 pixels	72 pixels/inch	
3C	Leica MZ16 light microscope (Germany)	10X magnification	Powerpoint	Cropping + grey-scale	1280x1024 pixels	72 pixels/inch	
4C-F + 5E+G	Olympus IX83 microscope with a Yokogawa scanning unit	60X/1.4 NA oil immersion objective	CellSens Dimension Software<br><br>ImageJ (color balance adjustment)	ImageJ:<br>- Color adjustment via ‘color balance function’<br>- Merge channels<br>- Transform from 8 bit to ‘RGB’<br>- Save as tiff	2048x2048 pixels	96 pixels/inch	Images taken as snapshots.<br><br>ImageJ info:<br>Resolution: 96 pixels per inch<br>Pixel size: 0.0104x0.0104 inch^2<br>Bits per pixel: 32 (RGB)
	Same	Same	Same	ImageJ:<br>- Split channels,<br>- Adjust color via ‘color	Same	9,23 pixels/inch (individual color + for merged images)	Images of multiple channels acquired at the same time.

				balance' function<br>- Merge channels<br>- Transform to 'RGB' (from 8 bit), - Save as tiff.			Image format: vsi<br>8-bit per channel (until 'transform to RGB')<br><br>ImageJ info:<br>- Resolution: 9.2308 pixels per micron<br>- Pixel size: 0.1083x0.1083 micron^2,<br>- Bits per pixel = 32 (RGB)
	Same	Same	Same	ImageJ (after deconvolution):<br>- Z-project (Max intensity)<br>- Split channels<br>- Save individual unedited image<br>- Adjust color balance (and save as an individual image)<br>- Merge channels<br>- Transform to 'RGB' (from 8 bit), - Save as tiff.	Same	9,23 pixels/inch (individual color + for merged images)	Images acquired as Z-stacks.<br><br>Non-deconvoluted image:<br>- Resolution: 9.2308 pixels per micron<br>- Pixel size: 0.1083x0.1083 micron^2,<br>Bits per pixel = 8 bit<br><br>Deconvoluted image:<br>- Resolution: 9.2308 pixels per micron<br>- Pixel size: 0.1083x0.1083 micron^2,<br>Bits per pixel = 8 bit<br><br>Merged image:<br>- Resolution: 9.2308 pixels per micron<br>- Pixel size: 0.1083x0.1083 micron^2,<br>Bits per pixel = 32 bit

Secondary antibodies:
F(ab')2-Goat anti-Mouse IgG (H+L) Cross-Adsorbed Secondary Antibody Alexa Fluor 568 (A11019)
Laser line: 568 nm
Common filter set: Texas Red
Excitation max: 578 nm

Emission max: 603 nm

F(ab')2-Goat anti-Mouse IgG (H+L) Cross-Adsorbed Secondary Antibody Alexa Fluor 488 (A11070)
Laser line: 488 nm
Common filter set: FITC
Excitation max: 490 nm
Emission max: 525 nm

Supplementary file: Table S2

Title of data:

Table of antibodies

Description of data:

A table containing the cellular target, Supplier and catalogue number of the antibodies employed during this study.

**Antibody name**	**Cellular target**	**Supplier, Cat. #**
Caspase-7	Full and cleaved Caspase-7 protein	Cell Signalling, #9492
Poly-ADP Ribose Polymerase (PARP)	Full and cleaved PARP protein	Cell Signalling, #9542
C/EBP homologous protein (CHOP)	Marker of ER stress	Cell Signalling, #2895
pSer807/811-Rb	Marker of proliferation	Cell Signalling, #9308
Akt	Akt (protein kinase B)	Cell Signalling, #2895
pSer473-Akt	Phosphorylated Akt (protein kinase B)	Cell Signalling, #4058
ERK	ERK	Cell Signalling, #4696
p-p44/42 ERK (Thr202/Tyr204)	Phosphorylated ERK	Cell Signalling, #9101
LC3B	Autophagy	Cell Signalling, #2775
γH2AX(Ser193)	DNA damage	Cell Signalling, (#2577)
p53	p53	Cell Signalling, 2524
p62	Autophagy	Abcam, #56416
TOM20	Mitochondria	Proteintech #11802-1-AP

PDI	ER	Thermo Fischer Scientific<br>#MA3-019
Giantin	Golgi apparatus	Enzo, (#ALX-804-600-C1100
LAMP-1	Lysosomes	Santa Cruz Biotechnology,<br>#20011
DCTN1	p150, loading control	BD Transduction Laboratories,<br>#610473
